# Transient binding sites at the surface of haloalkane dehalogenase LinB as locations for fine-tuning enzymatic activity

**DOI:** 10.1371/journal.pone.0280776

**Published:** 2023-02-24

**Authors:** Agata Raczyńska, Patryk Kapica, Katarzyna Papaj, Agnieszka Stańczak, Divine Shyntum, Patrycja Spychalska, Anna Byczek-Wyrostek, Artur Góra

**Affiliations:** 1 Tunneling Group, Biotechnology Centre, Silesian University of Technology, Gliwice, Poland; 2 Biotechnology Centre, Silesian University of Technology, Gliwice, Poland; KTH Royal Institute of Technology, SWEDEN

## Abstract

The haloalkane dehalogenase LinB is a well-known enzyme that contains buried active site and is used for many modelling studies. Using classical molecular dynamics simulations of enzymes and substrates, we searched for transient binding sites on the surface of the LinB protein by calculating maps of enzyme-ligand interactions that were then transformed into sparse matrices. All residues considered as functionally important for enzyme performance (e.g., tunnel entrances) were excluded from the analysis to concentrate rather on non-obvious surface residues. From a set of 130 surface residues, twenty-six were proposed as a promising improvement of enzyme performance. Eventually, based on rational selection and filtering out the potentially unstable mutants, a small library of ten mutants was proposed to validate the possibility of fine-tuning the LinB protein. Nearly half of the predicted mutant structures showed improved activity towards the selected substrates, which demonstrates that the proposed approach could be applied to identify non-obvious yet beneficial mutations for enzyme performance especially when obvious locations have already been explored.

## Introduction

Several naturally occurring proteins of viral, prokaryotic, and eukaryotic origins have been identified and repurposed for uses in the pharmaceutical, cosmetic, and food industries. However, their applications are often limited by enzyme activity, narrow substrate range, low stability, and loss of function in heterologous hosts [1]. To circumvent this problem, protein engineering attempts to develop new protein variants with better physiochemical and biological properties [2]. This process usually includes different genomic and post-genomic strategies that expand the functional ability and allow for the *in vivo* and *in vitro* synthesis of novel protein variants. One of the most widely used techniques in protein engineering is PCR-based random mutagenesis. This method can easily create a huge library of mutants that will be screened to identify the desired protein variants. However, in the absence of high-throughput screening tools, this process can be labour intensive, costly, and time consuming [3]. Recently, computer-aided molecular simulations have significantly benefited the field of protein engineering [4, 5]. Researchers can predict the significance of the introduced protein modifications by resolving the crystal structure of the target protein or by modelling possible structures based on their homologues. Additionally, the use of molecular modelling, molecular docking of a substrate or inhibitor, or dynamics simulations can facilitate the creation of an intentionally limited number of mutant libraries that can be screened for the desired properties following recombinant production [6].

The most widely targeted sites in protein engineering include the active site, its vicinity and other functional compartments, such as tunnels, allosteric sites, and cofactor sites. While the active sites of several proteins are located on the surface, over 60% of enzymes have a catalytic centre hidden inside their core [7]. These sites are connected to the surface of the protein by tunnels, which are used for the transport of substrates, products, ions, and water molecules. Enzyme and protein kinetic models, which are intended to better describe the phenomenon of ligand binding to enzyme active centres, suggest that tunnels may play a key role in enzymatic kinetics [7, 8]. In addition, it has also been suggested that ligand interactions with the enzyme surface are important [9–11]. These interactions can be useful in determining key surface amino acids that interact with the ligand on the protein surface and/or are involved in delivering ligands into the protein interior. Later on, they can also be used to identify potential sites on the protein surface that can be engineered to improve the activity or specificity of the enzyme. Such distal from the active site mutations have proved to cause changes of enzymes conformational landscape and therefore influence the enzymes activity [12–17]. One of the pioneering studies that demonstrates the potential association between surface residues and ligand binding involved the complete reconstruction of the benzamidine-trypsin binding process through molecular dynamics (MD) simulations. In this study, the authors identified several metastable states in the protein, and one of the transition steps was proposed as the rate-limiting step [18]. In addition, a study describing the relevant hot-spots associated with enzyme-substrate complex formation in dihydrodipicolinate synthase demonstrated that such key metastable binding intermediates can also be important for enzymes with surface-exposed active sites [19]. Furthermore, the importance of surface binding pockets was also highlighted in a recent nonequilibrium MD study, which revealed potential conformational changes of a binding site that resulted in transient pocket transformation [20]. In addition to the binding pockets, small potential binding sites called “cryptic sites” were shown to exhibit above average flexibility. In another study describing fatty acid binding to human serum albumin, a stochastic nature of ligand binding process was explored. Additionally, linear dependence of average time of insertion of the fatty acid on its distance from the binding position was shown [21]. The active site region vicinity was also explored in a study focused on MD and docking studies of HIV Integrase with 5CITEP inhibitor. The conducted analysis enabled recognition of a new and possibly important binding region on the surface lined with loop residues that were previously built-in [22]. These studies, however, focused mostly on their potential druggability, and therefore, their role in enzyme improvement remains largely unknown [23].

It is important to note that, although the previously mentioned studies utilise quite expensive computational methods, they were not applied for protein engineering purposes. Therefore, the aims of the current study are to integrate computational tools into the design of novel proteins and analyse the potential role of surface-positioned amino acids in protein stability and activity. Even a small improvement in the activity of a commercially-available enzyme can be highly valuable, and the rational-based approach could make such a design simpler and more cost-effective. Such a method could be of great value particularly when obvious locations have already been explored. We propose an approach which can deliver a small, smart library of potentially interesting mutants (~10 variants) rather than a set of specific positions that could be later validated to a high extent. As a model system, haloalkane dehalogenase LinB derived from the *Sphingomonas paucimobilis* UT26 was selected. It is one of the most comprehensively studied enzymes with a buried active site. The mechanism of catalysis of the LinB enzyme was described in 2003 [24]. In the years that followed, LinB was used as a model system for several studies: the modification of activity and selectivity of the enzyme by tunnel entrance engineering [25], quantitative analysis of substrate specificity [26], a combined theoretical and experimental study of the effect of partial closing of the entrance tunnel on the mechanism and kinetics of product release [27], protein-ligand binding study by CGMD simulations [28], stability improvement [29], *de novo* tunnel opening [30], enantioselectivity [31], and a recent study on the impact of tunnel mutations on enzymatic catalysis [32].

LinB is a relatively small enzyme forming two domains: the core and the cap domain linked together by the hinge. The active centre is located inside the pocket between these two domains and is connected to the protein surface by the main tunnel p1. The p1 tunnel is formed by two branches [30] and is used for substrate and product transportation. An accessory tunnel p2 is used to transport water molecules needed for the hydrolysis stage in the enzymatic reaction [33]. LinB exhibits moderate flexibility of its fragments that can adopt different conformations. The B-factor values determined for the cap domain suggest that this domain is significantly more dynamic compared to the core domain. Among them, the loop 144–148 located at the entrance to the main tunnel leading to the active centre was found to be the most flexible one [33]. It is believed that this region maintains the hydrophobic nature of the pocket and allows large substrates to enter the catalytic site [33, 34]. Due to the location of the active site, the rate-limiting step of the reaction can easily be modified by a single substitution. In the LinB wild type (WT), the hydrolysis of the intermediate alkyl limits the enzyme activity [24], while the introduction of a bulky amino acid close to the entrance of the p1 tunnel (LinB L177W) changes the rate-limiting step to the release of the product [27]. Interestingly, by separating the product and substrate transport pathways, it was possible to achieve the fastest haloalkane dehalogenase. This improvement was enabled by opening the additional new tunnel p3, which is perpendicular to existing ones due to three additional mutations: W140A, F143L, and I211L [30]. It is worth mentioning here that the mutant was selected from 12 distinct sequences derived from 36 positive candidates selected from 4140 colonies [30].

Here we use a theoretical approach to predict surface-exposed and potentially mutable residues of the LinB WT, with the aim of creating a small, smart library that can be verified experimentally. The substrates selected for the *in silico* studies include the following: 1-chlorohexane, 1-bromobutane, 1-iodopropane, 1,2-dibromoethane, 1,2-dibromopropane, bromocyclohexane, and chlorocyclopentane. These substrates represent both cyclic and aliphatic compounds of various chain lengths with different halogens and were specifically selected to check if and how these features affect the interaction of the substrate with the enzyme surface. MD simulations were performed with all selected substrates, and the ligand interactions which occurred with the enzymes’ solvent-exposed amino acids, led to the selection of positions that had the longest interaction time. Then, in order to examine the effect of substituting these key residues, we calculated their energetic contributions to the proteins stability using FoldX [35] to avoid unfavourable substitutions. Based on the energetically favourable mutations together with the hydrophobic and hydrophilic properties of the substituted amino acids, we chose the ten most promising mutations in six positions for further experimental analysis and proceeded with the mutants design, production, and purification. Their thermostability was tested using nano differential scanning fluorimetry (nanoDSF), and their activity was measured with two substrates. As a result of the study, among the ten designed mutants, eight were successfully produced, four had improved stability, five had improved activity towards chlorohexane and three towards bromocyclohexane. These results provide a near 50% success rate.

## Materials and methods

### Computational

#### Systems preparation

The X-ray crystallographic structure of haloalkane dehalogenase LinB from *Sphingomonas paucimobilis* UT26 (PDB ID: 1MJ5) [34] was downloaded from the Protein Data Bank [36]. Metal ions were removed manually from the crystal structure, and water molecules were saved. Additional water molecules were added as described previously [37].

The protonation states of titratable residues were determined using the H++ Server [38] at pH 8.6 (pH for experimental activity measurement). LeaP from AmberTools18 suite [39] was used to add counterions and immerse all models in a box of TIP3P water molecules. The substrates 1,2-dibromoethane, 1,2-dibromopropane, 1,2-dichloroethane, bromocyclohexane, chlorocyclopentane, 1-chlorohexane, 1-bromobutane, 1-iodopropane were downloaded from the ChemSpider database [Royal Society of Chemistry, ChemSpider database http://www.chemspider.com/, 2020, accessed March 2020]. Force field parameters and libraries for ligands (except 1-iodopropane) were generated using the R. E. D. Server [40] with Gaussian 09 [41] as QM program andRESP-A1 and AMBERFF10 as a charge model and force field, respectively. In the case of 1-iodopropane, the R. E. D. Server was also used, but the obtained parameters were not complete (the angle value H1-CT-I and its strength constant were missing). To obtain the desired values for 1-iodopropane, an output structure from the R. E. D. Server was optimised, and the frequencies were calculated. The size of the angle and force constant were read from the formatted checkpoint file (.fchk) in XYZViewer [Calistry XYZview. (online.xyz file format visualizer). http://calistry.org/calculate/xyzviewer, 2020, accessed April 2020]. The method used was described in [42]. AddToBox program [39] was used to randomly place four substrates around the protein at a distance of at least 5 Å from the protein and from each other. The procedure was run ten times to obtain ten different models for each of the selected substrates.

#### MD simulation

The Amber 18 package [39] was used to perform the simulation procedure of the LinB wild type (WT) using the ff14SB force field [43] in ten replicas for each simulated system. The minimization procedure consisted of 1000 steps, involving 500 steepest descent steps followed by 500 steps of conjugate gradient energy minimization with decreasing constraints on the protein backbone (500, 125, and 25 $\frac{kcal}{mol\times {Å}^{2}}$) and a final minimization with no constraints of conjugate gradient energy minimization. Gradual heating was performed from 0 K to 298 K over 20 ps using a Langevin thermostat with a temperature coupling constant of 1.0 ps in a constant volume periodic box. Equilibration and production were run using the constant pressure periodic boundary conditions for 2 ns and 100 ns, respectively, with a 2 fs time step. Constant temperature was maintained using the weak-coupling algorithm for 100 ns of the production simulation time with a temperature coupling constant of 1.0 ps. Long-range electrostatic interactions were modelled using the Particle Mesh Ewald method [44] with a non-bonded cut-off of 10 Å. SHAKE algorithm was used to maintain the geometrical constraints [45]. The coordinates were saved at intervals of 2 ps.

#### Ligand-protein interactions

The CPPTRAJ [46] software (mask function) from the AmberTools package was used to determine which amino acids in the protein interacted with the substrate during the entire simulation. The border distance, which is the contact distance between the enzyme and the substrate, was assumed to be 2.7 Å. If the position of the ligand was less than or equal to this distance from any amino acid atom, the program treated it as an interaction. The distance of 2.7 Å was chosen based on the LIGPLOT recommendations [47], which is a tool mainly aimed for calculating distances between molecules, but in static models, and was therefore not used in the analysis. Next, tables of interactions were obtained from the data generated in the previous step, using custom-written scripts by our group. The final results represented in a form of these tables are matrices (interactions with particular residues vs. frames of the MD simulations). Due to the limited number of the interactions of particular positions with the ligand in the course of simulations, the results of transient binding events appeared as sparse matrices, which are matrices where most elements are zero.

#### Clustering of the results

The clustering of results (sparse matrices) consisted of two stages that are presented in the workflow provided in **S1 Fig**. In summary, the first stage involved hierarchical clustering which was performed for each of the tables (representing one substrate from a given simulation replica) using scipy.cluster.hierarchy [SciPy.org, Hierarchical clustering (scipy.cluster.hierarchy)., https://docs.scipy.org/doc/scipy/ reference/cluster.hierarchy.html, 2020,accessed May 2020] library in python. Data was clustered with Jaccard metrics [48]. For further analysis, into account were taken clusters with a size of at least three amino acids obtained after the Jaccard index value cut off of 0.65. The groupings obtained in the first stage of clustering were called local clusters. In the second stage, the local clusters obtained from the previous step were subjected to hierarchical grouping with the same parameters. This procedure allowed for the identification of potential substrate transient binding sites in a global way and resulted in previously identified groups of amino acids being clustered separately for each ligand. As a result of these operations, the substrate-enzyme surface interaction sites were obtained for all seven of the tested ligands, representing the so-called global clusters. The time each ligand interacted with a given site was calculated from the median time of interaction with all local clusters, which was identified in the first stage of clustering.

#### Identification of the key interacting sites and amino acids

The next stage of the investigation was to identify key sites on the enzyme surface that may interact with the substrates and then subject them to further analysis (**S1 Fig**). For each substrate, the key clusters selected were those whose interaction time accounted for at least one third of the longest binding time to the enzyme surface, excluding those associated with the entrance to the main tunnel p1. This tunnel represents the main route for transporting substrates to the active site, and therefore amino acids that build up its entrance interact with substrates and products for a long time [28]. This elimination was executed to avoid losing relevant information about interactions with other surface amino acids. In this way, key amino acids interacting with surface-associated protein-ligand interactions were identified. For all amino acids included in a given cluster, the total length of time they interacted with the ligand was calculated and presented as the median interaction time associated with local clusters. Thereafter, the median from the substrate interaction times with all amino acids forming global clusters was calculated. All amino acids whose ligand interaction time was greater than or equal to the calculated median were classified as key amino acids and subjected to further analysis.

#### Stability prediction

The key amino acids obtained from the previous analysis were mutated into all possible nineteen amino acids using FoldX [35] (**S1 Table**). The BuildModel module of FoldX was used to introduce the selected mutation, optimise the structure of the mutants, and calculate the difference in the Gibbs free energy of protein folding between the WT and mutant variant. The lower the difference in energetic terms, the more stable the mutant variant should be.

#### Validatio

MD simulations with five of the selected LinB mutants: E78Q, A81D, A81N, T264M and A189F and substrates used for experimental studies: chlorohexane and bromocyclohexane were performed as previously for MD simulations with LinB WT. The MD simulations were calculated as ten replicas of 100 ns each. To minimise the influence of starting poses of substrate molecules on the detection of the binding sites, the initial positions of substrate molecules were copied from respective replicas of MD simulations of LinB WT with chlorohexane and bromocyclohexane. The interaction times were detected using Amber18 mask selection syntax. For validation purposes, the interaction times were not clustered as previously, but instead transient binding sites were the same as previously calculated for LinB WT with all seven substrates. The interaction times of residues in transient binding sites of LinB mutants and substrate molecules were compared to the interaction times of residues in binding sites of LinB WT with respective substrates. The results were presented in summary table (**S2 Table**) and on histograms (**S2–S7 Figs**).

### Experimental

#### Bacterial strains, plasmids, and growth conditions

*Escherichia coli* BL21(DE3) (New England Biolabs, USA) expressing T7 DNA polymerase was routinely grown in agar or liquid Luria-Bertani (LB) broth at 37°C. Where necessary, growth media were supplemented with 100 μg/mL ampicillin (Sigma-Aldrich) and 100 mM IPTG (Sigma-Aldrich). Recombinant plasmids containing either the wildtype (WT) or mutated LinB genes of *Sphingomonas paucimobilis* were designed *in silico* and synthesised by Biomatik (USA). *E*. *coli* BL21(DE3) and the pET-21b(+) plasmid (Biomatik, USA) were used for the overexpression of the WT and mutant LinB proteins. All bacterial strains and plasmids used in this study are listed in **S3 Table**.

#### Expression and purification of His-tagged LinB wildtype and mutant proteins

Recombinant pET-21b(+) plasmids were individually transformed into *E*. *coli* BL21(DE3) by heat shock as previously described [49]. Transformed bacterial cultures were grown in a 2.5 L Ultra Yield™ Flask containing 250 mL of EnPresso B B11001 medium (Enpresso GmbH, Germany) supplemented with ampicillin (100 μg/mL) and Antifoam 204 (Sigma-Aldrich, USA). Protein expression was induced according to the protocol supplied by the manufacturer with the IPTG final concentration of 100 mM. Bacterial cells were then disrupted by incubation with lysozyme and sonication using the Vibra-Cell™Ultrasonic Liquid Processor (Sonics, USA), and cell debris were removed by centrifugation. The resulting cell extract was subjected to Ni(II)-chelate affinity chromatography using HiTrap® IMAC Fast Flow column (5 mL, 16 mm GE Healthcare) to purify the C-terminal 6xHis-tagged proteins. Proteins were eluted with imidazole by gradient elution with a flow rate 5 mL/min. Dialysis was performed at 4°C for 18 h in potassium phosphate buffer (50 mM, pH 7.5) using the MEMBRA-CEL**®** dialysis tubing RC, diameter 16 mm, MWCO 3500 Da (Serva, Germany). Thereafter, the water from the samples was removed by lyophilization using laboratory freeze dryer ALPHA 1–2 LD plus (Martin Christ, Germany). The proteins were then dissolved in deionised water, and the concentration of the purified proteins was determined using Protein Quantification Kit-Rapid (Sigma-Aldrich, Germany) according to the manufacturer’s instructions.

#### Electrophoresis in SDS-polyacrylamide gel

25 μg of the proteins were mixed with a sample loading buffer containing 5% 2-mercaptoethanol. The samples were thermally denatured (5 min, 95°C) and run on polyacrylamide gel (90 min, 120V, stacking gel: 5%, resolving gel: 15%). The gel was stained overnight with Coomassie brilliant blue R-250, destained (10% acetic acid, 40% ethanol), and the image was captured with the G:Box transilluminator (Syngene, UK).

#### Western blot analysis

100 ng of the proteins were loaded onto the SDS-polyacrylamide electrophoresis gel (90 min, 120V, stacking gel: 5%, resolving gel: 15%). Next, the proteins were transferred onto a nitrocellulose membrane (30 min, 4°C, 350mA in a transfer buffer: 5mM Tris, 40mM glycine, 0.1% SDS, 20% methanol). After the transfer, the nitrocellulose membrane was blocked (45 min, 4°C with 5% skim milk in TBST buffer: 10 mM Tris-HCl pH 7.5, 150 mM NaCl, 0.05% Tween 20). Next, the membrane was rocked with the primary antibodies His-Tag (D3I1O) (1:1000 dilutions, Cell Signaling Technology, USA) at 4°C. Afterwards, the membrane was incubated with the appropriate HRP-conjugated secondary antibodies (dilutions 1:1000, Cell Signaling Technology, USA). Proteins were visualised with the Immobilon® Western Chemiluminescent HPR Substrate (EDM Millipore Corporation, USA) and analysed using the G:Box transilluminator (Syngene, UK).

#### Nano differential scanning fluorimetry

Thermal unfolding of the proteins was measured during nano differential scanning fluorimetry (nanoDSF) experiments with the Prometheus NT.48 (NanoTemper Technologies, Munich, Germany) instrument. In summary, 10 μL of the protein samples with a concentration of 1 mg/mL were loaded into Standard Prometheus NT.48 capillaries. The melting curves of the proteins were obtained with a temperature gradient from 20°C to 95°C with a 0.5°C/min stepwise increment. The shift in intrinsic tryptophan fluorescence was measured at emission wavelengths of 330 nm and 350 nm. PR.ThermControl software was used to acquire and analyse the data.

#### Far-UV circular dichroism spectropolarimetry (Far-UV CD)

The Far-UV CD spectra of the proteins with a final concentration 150 μg/mL in a 50 mM potassium phosphate buffer (pH 7.5) at 20°C were recorded using the Jasco J-815 spectrometer. The data was collected from 185 to 260 nm with a 100 nm/min stepwise increment using 0.1 cm quartz cuvette with a 1 s response time and 2 nm bandwidth. The results were recorded as the average of 10 individual scans.

#### Enzymatic reactions and GC analysis

Before starting the reaction, a mixture of glycine buffer (100 mM, pH 8.6) and the appropriate substrate (1-bromocyclohexane, final concentration: 8.1 mM or 1-chlorohexane, final concentration: 7.2 mM) was incubated in a Screw Top Mini Flask with Hole Cap and Septum (Sigma-Aldrich, USA) on a shaker for 30 min (37°C, 275 rpm). Thereafter, the protein was introduced to the mixture, and the reaction was carried out for 20 min. The samples were withdrawn from the enzymatic reaction mixture every 4 min using a syringe and were mixed with the same volume of a mixture of methanol and IS (1,2-dichloroethane) in a 1000:1 ratio (v:v) to quench the catalyst. The increase in product was then analysed by gas chromatography (Hawlett Packard 5890 Series II GC, USA) on the 30 m x 0.32 mm LD (film thickness 0.5 μm) DB-WAXETR column (Agilent, USA). The concentration of the compounds was calculated based on prepared calibration curves. Conditions of the analysis were as follows: cyclohexane: oven 150°C, injector 140°C, detector 260°C; hexane: oven 120°C, injector 110°C, detector 260°C; air to hydrogen ratio: 10:1, gas flow rate: 1.3 mL/min in 30°C, split: 1:100. The enzymatic reactions for each enzyme and each substrate were prepared in at least 3 replicates, and the final enzymatic activity was presented as an average value.

## Results

### Computation

The aim of the *in silico* part of the study was to provide a short list of residues to build a smart library of mutants with potentially modified enzyme activity. The additional criteria were to select residues that are distant from the known functional residues, including active site residues and residues building the functional tunnels, such as the main transport tunnel p1 (which provides access to the active site) and the water transporting tunnel p2. Our strategy was based on the identification of transient binding sites on the protein surface and on the selection of amino acids that contribute to short-lived interactions. The overview of the workflow is presented in the scheme (**S1 Fig**).

Ten replicas of the molecular dynamics (MD) simulations of the LinB enzyme (PDB ID: 1mj5) were run with one of the seven substrates: 1-chlorohexane, 1-bromobutane, 1-iodopropane, 1,2-dibromoethane, 1,2-dibromopropane, bromocyclohexane, and chlorocyclopentane. The number of ligands in each MD run was calculated to mimic the substrate concentration in reaction conditions (see Materials and methods section). The preliminary analysis of the interactions was performed with normal mode and PCA analysis. This qualitative analysis showed differences between maps of the normal modes obtained for each system and significant changes of the normal mode distribution caused by the presence of the substrates (data not shown). Based on this, we speculated that the substrate interactions with the protein surface can modify protein dynamics, and thus long-range interactions might modify enzyme properties.

#### Transient binding sites

**Fig 1:**
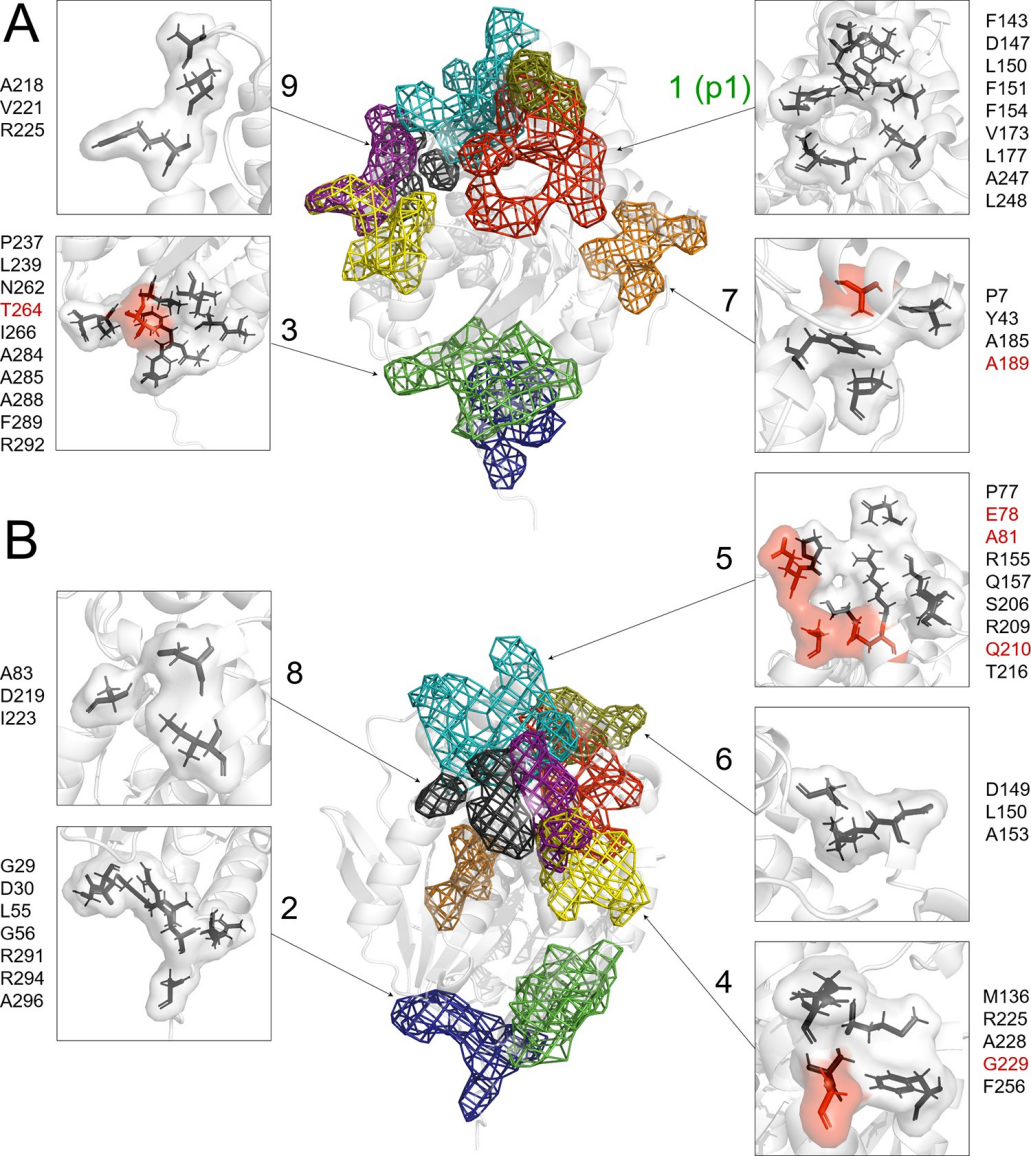
Structure of haloalkane dehalogenase LinB. LinB shown as a white cartoon representation with marked transient binding sites presented as a coloured mesh. LinB is shown in two orientations: (A) and (B). The side panels show zoomed-in views of transient binding sites along with their building residues shown as black sticks. Residues associated with a given transient binding site are indicated on the sides. Residues that were chosen for substitution are shown as red sticks and are labelled in red. Signatures above the arrows include the identifier of the transient binding site.

In order to provide quantitative analysis, a precise map of interactions was built. Interactions between substrates and the enzyme during MD simulations were detected using Amber18 mask selection syntax [39]. As a result, sparse matrices of transient binding events were obtained, where rows and columns represent the residue number and the number of frames of the simulation, respectively. Residues of LinB WT to which ligand molecules were bound were investigated and clustered into transient binding sites at the surface of the protein using hierarchical clustering methods. The clustering was conducted in two stages: (1) local and (2) global. In the first stage, clustering of LinB WT amino acids interacting with the substrate within one replica of the simulation was performed (**S8–S14 Figs**). As a result, interaction maps for each substrate with LinB WT were obtained and provided information about the total length of interactions and about each amino acid contribution to particular substrate binding. Based on these results, in the second stage, we built the global interaction map by clustering groups of amino acids interacting with the ligand for ten replicas of the simulation. Then, we identified nine transient binding sites, and obtained a list of potential amino acids aimed for further analysis (**Fig 1**). For MD simulations of LinB mutants (E78Q, A81D, A81N, T264M and A189F) with substrates (chlorohexane and bromocyclohexane), the interaction times were not clustered. For analysis of MD simulations with the mutants were used the same nine transient binding sites, as were calculated for MD simulations with LinB WT.

The global interaction maps were characterised using two main factors: the location and cumulative time of substrate interaction. We observed a large variance in the number of the interaction sites, from only 2 (1-bromobutane) to 7 (1-chlorohexane) (**S8–S14 Figs**). The number of identified sites correlated neither with the size of the analysed substrate nor with the type of halide ion present in the substrate. Since the p1 tunnel is considered the main access pathway to the active site of LinB, we expected to identify interactions of all substrates with the residues building the p1 tunnel entry. Indeed, the transient binding site 1 corresponds to the entrance of the p1 tunnel. Each of the analysed substrates was found to interact with the p1 tunnel entry, and in most cases, the cumulative time of interactions with this site was the longest. The only exceptions were for 1,2-dibromoethane and 1,2-dibromopropane, for which the longest interaction times were with the site located close to the N-terminal of the LinB protein (48 ns vs. 54 ns and 62 ns vs. 47 ns, respectively). The p1 tunnel entry was not included in our analysis because this site has been extensively studied [25, 27, 50]. Moreover, residues at the p1 tunnel entry were interacting with ligands during substrate entry to the active site, which would bias the results in relation to residues from other transient binding sites. The analysis also showed that most of the substrates interacted with the transient binding site 4, which is separated from the entrance of the p2 tunnel by an α9-helix. The only exception was chlorocyclopentane, which interacted with 5 different sites but not with transient binding site 4. Interestingly, transient binding site 8 was found to be located at the entrance of the *de novo* created p3 tunnel [30]. In our study, only a single transient binding site was identified exclusively on the cap domain (6). The majority of the transient binding sites were identified in the core domain (2, 3, 8, 9) or were formed by the amino acids of both domains (1, 4, 5 and 7). Two of the transient binding sites, 9 and partially 4, are located close to the "hinge" joining both domains in the region of the α4 and α8 helices.

#### Key amino acids

The transient binding sites that were identified as interacting with particular ligands were extensively analysed. The focus was on the cumulative length of interactions with sites and with particular amino acids along with their chemical properties. The aim of the analysis was to identify the most important sites and amino acids whose substitution could potentially have the highest impact on the physicochemical composition of the transient binding site. Our results show that the cumulative time of the interaction varied significantly from 9 to nearly 100 ns depending on the substrate and site (**Table 1, S8–S14 Figs).** For each substrate, the selected clusters were those whose interaction times were at least one third of the longest interaction time with the enzyme surface and were not associated with the entrance to the main tunnel p1. Next, key amino acids constituting a transient binding site were chosen. In this way, the interaction time with ligands was defined as the median of all interaction times of a given residue in all replicas (local clusters that were considered when constructing global clusters). Thereafter, we calculated the median interaction time of each substrate with all amino acids of global clusters. The amino acids with interaction times greater or equal to the calculated median were classified as key amino acids and were further investigated.

**Table 1:** Cumulative time of the measured interactions of particular substrates with transient binding sites.

	Cumulative time [ns]						
Transient binding site	1CH	1BB	1IP	12DBE	12DBP	BCH	CCP
1	73.9	39.7	28.3	48.0	47.1	82.9	54.8
2	21.3	-	-	-	-	-	-
3	23.0	-	26.4	54.4	62.4	68.9	25.4
4	59.9	17.2	9.3	26.2	20.1	45.8	-
5	96.6	-	-	-	-	32.8	38.2
6	-	-	-	-	-	16.5	-
7	32.0	-	-	-	-	-	-
8	-	-	-	-	-	-	20.5
9	-	-	-	-	-	-	15.7

1CH: 1-chlorohexane, 1BB: 1-bromobutane, 1IP: 1-iodopropane, 12DBE: 1,2-dibromoethane, 12DBP: 1,2-dibromopropane, BCH: bromocyclohexane, CCP: chlorocyclopentane. The green shading corresponds to the value of cumulative time.

#### Smart library

**Fig 2:**
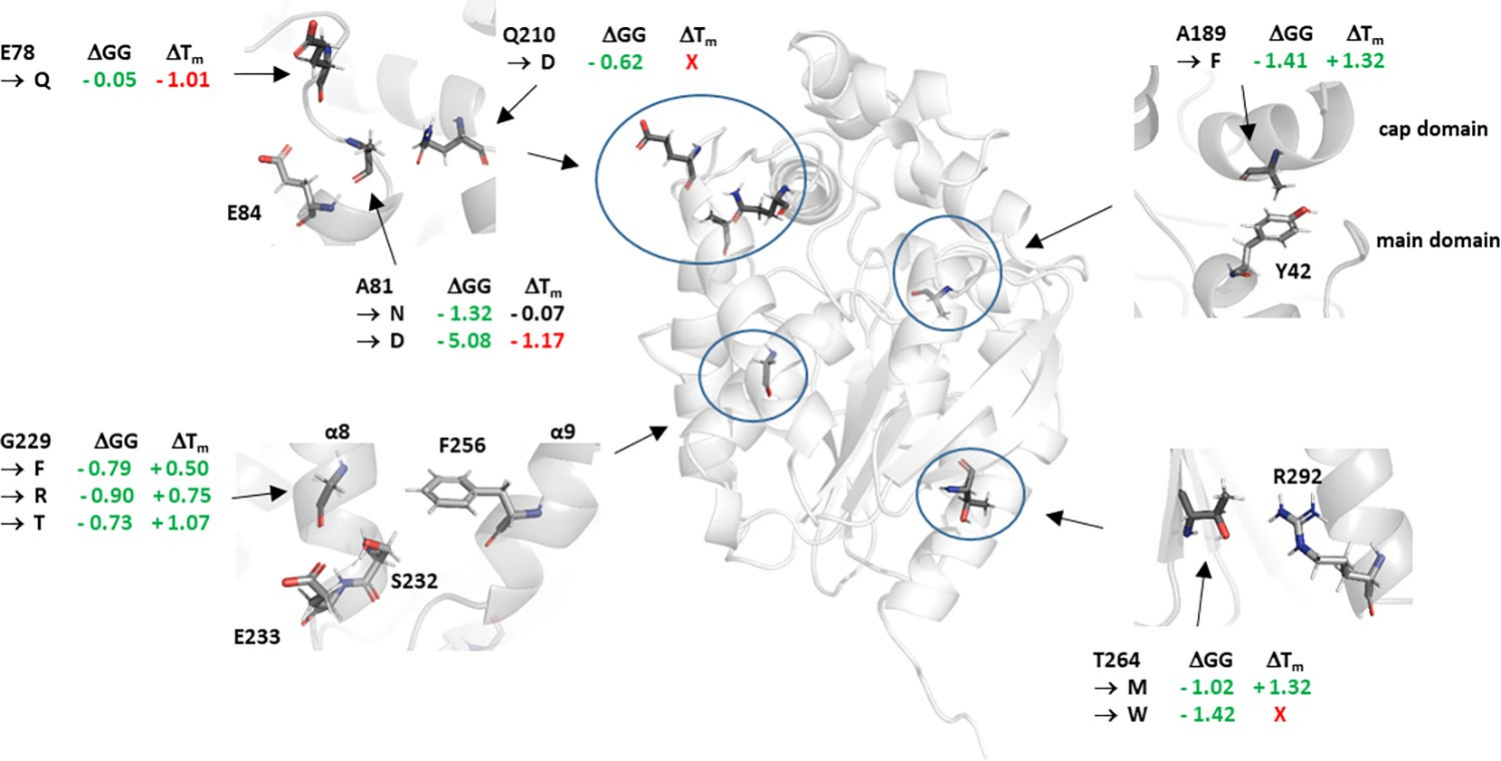
A comparison between experimentally-determined melting temperature of LinB mutants (ΔT_m_) and the predicted changes in the Gibbs free energy (ΔGG). Green colour indicates increase in stability, and red colour indicates decrease. Red X indicates failure during mutant production. The positions of the zoomed-in regions were rotated to facilitate analysis of crucial interactions.

The list of the key amino acids from the previous step was considered as a preselection of residues that can be considered for mutation. Next, all potential mutations to the wild type structure of haloalkane dehalogenase LinB were proposed with FoldX [35]. As a result, differences in the Gibbs free energy of protein folding with respect to the wild-type protein were obtained (**S1 Table**). The most promising mutations were chosen based on the following: (1) interaction time with ligands–that way important residues in enzyme-ligand interactions would be chosen; (2) substantial energetic decrease–so that mutations would not interfere with the stability of the protein structure according to the differences in Gibbs free energy of protein folding; and finally (3) the type of mutated amino acid–so that the character of the selected residue could be modified. Using the previously mentioned parameters, we selected six residues (E78, A81, A189, Q210, G229, T264). Some of these residues were replaced with more than one different amino acid, and in the end, ten mutants were selected (E78Q, A81D, A81N, A189F, Q210D, G229F, G229R, G229T, T264M, T264W) (**Fig 2)**. This extremely small library of single substituted variants was used to experimentally examine and validate the potential role played by surface residues in fine-tuning activity of the enzyme.

#### Validation

Interaction times of LinB mutants residues and two substrates: chlorohexane and bromocyclohexane were compared to interaction times of LinB WT and respective substrates (**S2 Table** and **S2–S7 Figs**). The results show substantial change (over 50%) of the interaction time with the mutation-carrying binding site. For most of the other sites such change was not substantial (below 20%). However, in case of the A81 substitution for aspartic and glutamic acid, despite decrease of the interaction time with the mutation-carrying binding site (5^th^), the large increase of the interaction time with the 3^rd^ binding site was observed. Similar situation was found for other substitution in 5^th^ binding site–the E78Q, which also affect cumulative time of the interaction with 3^rd^ binding site. Interestingly, binding sites 3 and 5 are on the opposite sides of the protein.

### Experimental

#### Production, purification, and basic analysis

During the experiments, we obtained the wild type and eight out of ten proposed mutated proteins with a yield between approximately 20 and 30 mg/l. The remaining two mutants (Q210D and T264W) were not successfully expressed (**S4 Table**). The purity of the expressed enzymes was confirmed by SDS-PAGE and Western blot analyses (**S9 and S10 Figs**). The proper folding of the purified enzymes was analysed using circular dichroism. According to our predictions, most of the mutations did not significantly alter the secondary structure of the protein (**S11 Fig, S4 Table**). A statistically significant difference was observed only for the G229F mutation, where we noticed a large decrease in the percentage of α-helices in the structure of the protein.

#### Stability and activity

The mutant proteins were further characterised based on their thermal stability and enzymatic activity. The thermal stability was analysed using the nanoDSF method, which measures the protein melting temperature (T_m_) (**Fig 2, S12 Fig)**. All analysed mutant proteins with a substitution in the G229 position and also the A189F mutant showed an increase in melting temperature relative to the WT (by 1.1% for G229F, 1.6% for G229R, 2.3% for G229T and 2.9% for A189F). On the other hand, the mutations A81D and E78Q decreased the T_m_ value by 2.5% and 2.2%, respectively. Nevertheless, the highest observed deviation was still quite small and did not exceed 1.5°C. Two of the introduced mutations, A81N and T264M, did not cause any significant changes in the protein stability.

**Fig 3:**
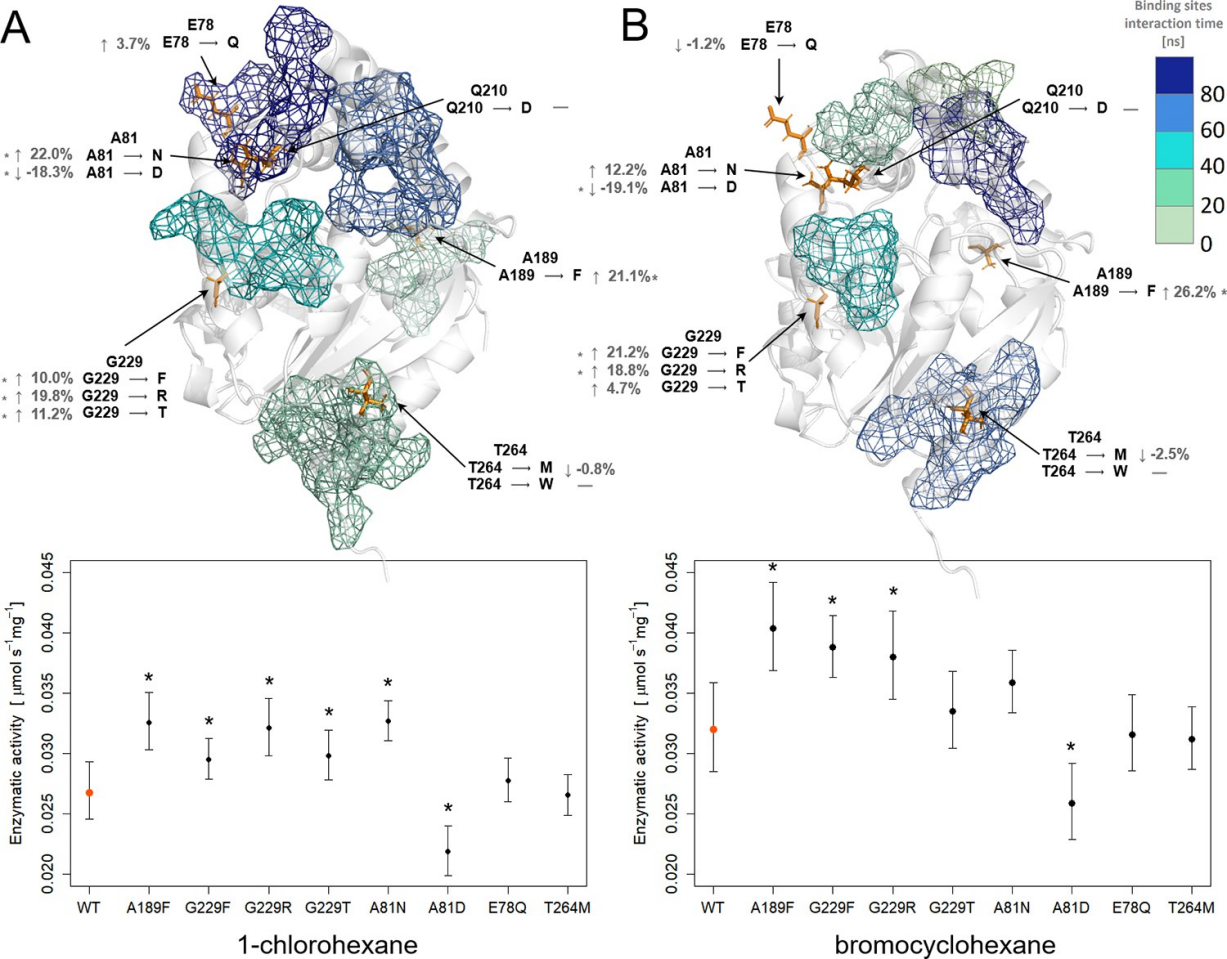
Activity of the LinB mutants determined experimentally in the reaction with (A) 1-chlorohexane and (B) bromocyclohexane along with transient binding sites calculated *in silico* for respective substrates. Mutated amino acids shown as orange sticks. Transient binding sites shown as mesh coloured according to length of interaction time with a selected ligand. Information on the changes in activity measured experimentally (in grey) of a selected mutation (in black). The plots underneath show forecasted mean values and associated confidence intervals of the forecasted values for experimental activity measurements of protein with (A) 1-chlorohexane and (B) bromocyclohexane. The asterisk above the point indicates a statistically significant difference in WT (red point) activity relative to the mutants.

Next, the specific activity of the produced variants towards 1-chlorohexane and 1-bromocyclohexane was analysed and compared to the activity of the WT **(Fig 3)**. These two substrates were selected due to the longest cumulative time of the substrate-surface interaction. The observed increase in stability of all G229 mutants and the A189F mutant was accompanied by a concomitant increase in enzyme activity. The most significant improvement was observed for hydrolysis of bromocyclohexane by the A189F mutant (26.2%). The increase in enzyme activity for both tested compounds was also observed for the almost stability-neutral A81N substitution. In contrast, the activity of the T264M mutant, which represents one of the most stable enzymes engineered in this study, remained largely unchanged. The highest drop in activity was observed for the A81D mutant. Interestingly, this mutant had the worst observed stability prediction (calculated ΔGG gain of -5.08 kcal/mol, measured drop in T_m_ from 46.03°C to 44.86°C).

## Discussion

The LinB dehalogenase has previously been used as a model enzyme in many studies aiming to use protein engineering to enhance beneficial properties. Among the most impressive were improvements of this enzyme’s activity to a previously unseen level [30]. Due to the active site location, most of the modification was related to the enzyme’s tunnels (closure, modification, or opening a new one) and engineering of the gates that regulate access to the catalytic triad [25, 27]. Such a strategy is considered safer since the residues that are directly involved in reaction catalysis are untouched; however, it is difficult to predict the direction of the changes. A pioneer study in 2007 by Silberstein and co-workers [10] used a solvent-mapping approach–which included small organic molecules, 1,2-dichloroethane, and halide ions–to map the potential surface binding sites on the surface of DhlA, DhaA, and LinB dehalogenases. They identified significantly larger and more solvent-accessible binding sites in LinB and DhaA in comparison to DhlA and rationalised that the more open active site eliminates the need for intermediate binding sites on surface. Despite the final conclusions, they identified three transient binding sites on the LinB surface: at the active site, at the entrance of the p2 tunnel, and close to the hinge region (corresponding to our transient binding site 5). The tunnel mouth of the main entrance was intensively investigated by mapping the ionic spatial distribution during molecular dynamics simulations. The significance of the detected interactions was verified experimentally and proved the importance of the tunnel mouth composition for catalysis [50]. The same study also demonstrated that cations affect enzyme activity by modulating substrate inhibition [50]. A year later, Negami *et al*. [28] used coarse-grained molecular simulations to describe interactions of LinB with 1,2-dichloroethane. The study identified two highly-populated sites (S1, S2) and two additional ones (S3, S4). Interestingly, two of the sites were also identified and investigated in our current study. They include the S1 site, which corresponds to transient binding site 1 and the entrance to the main tunnel, and the S3 site, which corresponds to transient binding site 8 (the site opened during *de novo* p3 tunnel design). Even though some distant from p1 tunnel entrance sites were predicted as potentially important, experimental verification was provided solely for mutations in the regions directly associated with transport of ligands to/from the active site.

In enzymes other than the LinB, mutations distal from the active site have proved to regulate enzyme by altering the conformational landscape and inducing the desired change in function [12]. Targeting residues distant from the active site, which may seem like non-essential residues for the physiochemical properties of enzymes, is a tempting way of fine-tuning the method of protein engineering. It can be used to improve enzyme activities since even small improvements can significantly promote enzyme usage as a green and economically beneficial solution, in particular when obvious sites have already been explored. Recent advances in computational methods, including methods for selection of key residue substitutions or solvent distribution analysis, have significantly improved protein design; however, the rational selection of such residues can still be a challenging task [5, 51].

The approach used in our current study focused on short-term interactions of particular substrates with the residues on the protein surface that are distant from the major substrate and product transport pathways including known gating residues. We wanted to verify if modifications in aforementioned residues involved in short-term interactions might influence enzyme activity. We aimed to provide a pipeline to design a small library of potentially beneficial mutations by applying combined *in silico* methods and experimental verification. Such a method could be used for enzyme fine-tuning, when mutations in obvious sites such as active site and surrounding, allosteric sites and transport pathways have already been explored. The choice of the residues for mutations was supported by modelling interaction times in MD simulations of the protein in the presence of substrates, followed by analysis of the effect of substitution on protein stability based on calculations of the differences of Gibbs free energy of protein folding and finally the substantial difference in physicochemical properties. The first of these steps significantly reduced the number of residues that need to be validated, whereas the second narrowed the potential mutations to those that would not destabilise the protein. The final step of the selection aimed to potentially provide the largest effect on the chemical nature of the transient binding sites; therefore, we preferred exchanging aliphatic residues for aromatic, charged for uncharged, positively charged for negatively charged. In the end, such a strategy allowed us to drastically decrease the number of variants that would be experimentally studied from 130*19 (which could be unrealistic even for high-throughput screening techniques) to only ten with an overall success rate of around 50% for improving enzyme activity and stability. We need to emphasise here that the improvement in protein stability was not the main goal of this study. According to Goldenzweig et al. [52], the stability of the protein can influence the efficiency of production because proteins with low stability usually do not fold properly. Because of that, we were selecting the neutral or beneficial mutations solely to limit the number of poorly expressed and misfolded variants. Indeed, we have observed a correlation between thermal stability and protein yield.

A close inspection of the selected positions allows us to rationally explain the observed improvement or disagreement (**Fig 2**). In the case of substitutions of a nonpolar amino acid for a polar one (mutations G229R, G229T, A81N, and A81D), the improvement in stability was small and only observed for two of them (G229R and G229T). The substitution of glycine for arginine (mutation G229R) could lead to formation of additional ionic interactions with the glutamic acid E233 and dipole-dipole interactions with serine S232. In addition, G229 is located on the α8-helix and faces the F256 located on α9-helix. It is possible that introducing both mutations may tighten interactions between the both helices. The observed improvement in protein stability could arise from the structural limitations; some of the hydrophobic amino acids cannot be placed inside of the enzyme surrounded by aqueous solution, and hence are left on the surface, thus decreasing the overall stability of the protein. Substitution of these amino acids for hydrophilic ones allows for the formation of more favourable hydrogen bonds and the improvement of stabilizing interactions in the microenvironment of the exchanged amino acid [53]. Another possible explanation of the increased stability of G229X mutants could be the decrease in the chain entropy of the unfolded state of the protein, caused by replacement of G with an amino acid containing a β-carbon [54]. The influence of the mutations A81X on protein stability can be associated with the properties of the inserted amino acid. In the case of substitution of A81 for asparagine, we did not observe any statistically relevant change in the protein. However, substitution of the same amino acid position A81 for aspartic acid lowered the stability of the LinB enzyme. This effect could possibly be caused by the proximity of this amino acid to two glutamic acids: E84 and E78. The addition of another positively charged amino acid might have formed undesirable ionic interactions, causing repulsion of all three amino acids and hence a decrease in thermal stability of the whole protein. We also observed improvement in thermal stability in LinB mutants with a substitution of a non-polar, small amino acid for a non-polar, bulky one (mutations A189F and G229F). The probable cause could be the formation of additional π-stacking interactions between the aromatic rings of F189 with Y42 and of F229 with F256, which stabilise the protein. The substitution of a polar amino acid for a non-polar one was performed only for the T264M mutant, for which we did not observe any significant change in thermal stability. Additionally, in the case of the E78Q mutant, we substituted a charged glutamic acid for its amide counterpart, glutamine. This modification drastically reduced the thermal stability of the protein, which can be explained by the decrease in hydrophilicity of the residue. Furthermore, the mutation may have disrupted the hydrogen bonds between this amino acid and the solvent.

The main goal of our study was to see if we could improve the activity of LinB by modifying residues distant from the active site and known transportation pathways, and seemingly non-essential residues. We need to empathise here that the goal of this study was not to solely verify the effects of modelling interaction time and check whether it could provide a useful way to predict beneficial substitutions. In order to verify such a hypothesis, we would have to choose the amino acids interacting with the ligands for the longest time—the gating residues or tunnel bottlenecks, whose mutation would most probably change enzyme’s performance to a much higher extent than the mutations that we proposed. Conversely, we assumed that modification of the residues involved in short-term interactions with substrates can have an influence on the overall activity of the enzyme. This is an almost unexplored field in protein engineering; however, several of the aforementioned studies were already pointing to the potential importance of cryptic sites for enzyme inhibition or kinetics. Even though substrate access is not considered as a rate-limiting step for LinB catalysis, we were able to observe over 20% improvement in the specific activity of some variants. Due to the indispensable role of the p1 tunnel, all residues contributing to the p1 tunnel entry, which represents a predominantly surface-exposed transient binding site, were omitted from our study. From other transient binding sites, we have selected for mutating one residue at sites 3, 4, and 7 and two residues at site 5. Nearly all tested compounds were able to bind to the transient binding site 3, which is located close to the N-terminal loop and far from any important locations within the enzyme. However, the T264M mutation at site 3 did not cause any statistically significant changes in the enzymatic activity. Similarly, site 4 was also shown to interact with most substrates. We analysed three variants carrying a mutation in the G229 position, and all had a significant effect on the enzyme activity, with up to 21.2% for G229F in the case of hydrolysis of 1-bromocyclohexane. We can only speculate that improvement of the activity is related to the relatively close position of the p2 tunnel entrance. The G229 is located on the α8-helix and faces the F256 residue located on the α9-helix. This neighbourhood can explain the observed stability gain for all constructed mutants. Moreover, stronger interaction between two helices may possibly influence the exit of the p2 tunnel, which is located nearby, and, as a result, may also modify water access to the active site.

The two substrates used in this study were found to interact with the transient binding site 5, constituted mostly from cap domain residues. For chlorohexane, the cumulative interaction time was comparable with the longest time observed for p1 tunnel entry, whereas for bromocyclohexane, the cumulative interaction time was over two times shorter. We analysed three different mutations on this site (A81N, A81D and E78Q), and the results were residue specific and no correlation between activity and cumulative interaction time was observed. Such lack of correlation should not be surprising since the interaction time was solely used as a preselection criterium. We averaged the interaction times with all the analysed substrates and what is more, we omitted the obvious sites on the surface that were associated with substrate and product transport pathways and had the longest interaction times. For example, for E78Q, we did not observe significant changes in the activity, but in the case of mutation A81D, we noticed a decrease in the enzymatic activity for both substrates (18.3% for 1-chlorohexane and 19.1% for 1-bromocyclohexane). In the case of the mutation A81N, we observed an increase in the enzymatic activity for 1-chlorohexane of up to 21.8%. It is worth emphasising that, for most of the mutants analysed in this study, we observed the same effect on enzymatic activity for both tested substrates (either an increase or a decrease in activity). It is possible that a change in the enzymes activity is associated with modifications in the flexibility of the cap domain (the site 5 is located close to the hinge region) or with long-range interactions involving halide stabilising residues (the W109 residue is facing site 5). The flexibility of the cap domain can be an essential factor for bulky substrate decomposition. The mechanism of bulky substrate entrance was recently described in the closely-related DhlA enzyme [55]. The highest improvement in protein activity was observed in a mutation located on site 7, where short-term interactions were exclusively detected with 1-chlorohexane. Surprisingly, the activity of the A189F mutant increased not only for 1-chlorohexane (by 21.4%) but also, and even more significantly, for 1-bromocyclohexane (26.2%). The A189 residue is located in the cap domain, and it is facing the Y42 residue located in the main domain. It is therefore possible that the introduced A189F mutation might strengthen contact between the cap and main domain in this region. This effect can be compensated by being in proximity and thereby affecting the entrance to the p1 tunnel.

In summary, in our work we aimed to select residues which can be promising for fine-tuning of enzymes when obvious sites were already explored. Therefore, we focused on nonobvious residues located on the protein surface but not in the proximity of main pathways of substrate and product transport. We proposed a pipeline for small library selection and we narrowed the number of residues preselected from 130 solvent-exposed residues to only six. We then applied substitutions by altering the type of amino acid in the chosen six sites–so that the character of the selected residue could be modified. In the end, we proposed a small library of ten LinB single-point mutants. We produced eight out of ten redesigned enzymes and verified their thermostability and activity. Five of the produced mutants showed significantly higher activity towards 1-chlorohexane and three towards bromocyclohexane. One of the mutants had significantly lower activity to both of the tested substrates, which also indicates an important residue that was substituted. We were able to obtain an almost 50% success rate and for some of the mutants we observed over 20% improvement in the specific activity of some variants, even though substrate access is not considered as a rate-limiting step for LinB catalysis. Our findings suggest that substituting key surface amino acids that interact with the ligand on the protein surface could be used for enzyme fine-tuning, especially when traditionally considered hot-spots were already considered, as even a small improvement in the activity of an enzyme can be highly valuable, and the rational-based approach could make such a design simpler and more cost-effective.

## Supporting information

S1 Fig The overview of the workflow performed to select a small library of the mutants. (TIF)

S2 Fig Histogram of LinB WT interaction times of residues with substrate: A) chlorohexane, B) bromocyclohexane. The interaction times were a sum of interactions of all 10 replica 100 ns simulations of LinB WT with a given substrate.
(TIF)

S3 Fig Histogram of LinB E78Q interaction times of residues with substrate: A) chlorohexane, B) bromocyclohexane. The interaction times were a sum of interactions of all 10 replica 100 ns simulations of LinB E78Q with a given substrate.
(TIF)

S4 Fig Histogram of LinB A81D interaction times of residues with substrate: A) chlorohexane, B) bromocyclohexane. The interaction times were a sum of interactions of all 10 replica 100 ns simulations of LinB A81D with a given substrate.
(TIF)

S5 Fig Histogram of LinB A81N interaction times of residues with substrate: A) chlorohexane, B) bromocyclohexane. The interaction times were a sum of interactions of all 10 replica 100 ns simulations of LinB A81N with a given substrate.
(TIF)

S6 Fig Histogram of LinB T264M interaction times of residues with substrate: A) chlorohexane, B) bromocyclohexane. The interaction times were a sum of interactions of all 10 replica 100 ns simulations of LinB T264M with a given substrate.
(TIF)

S7 Fig Histogram of LinB A189F interaction times of residues with substrate: A) chlorohexane, B) bromocyclohexane. The interaction times were a sum of interactions of all 10 replica 100 ns simulations of LinB A189F with a given substrate.
(TIF)

S8 Fig 1-chlorohexane interaction map with LinB surface. For each of the binding sites a histogram of interactions with particular residues and the cumulative interaction time (CT) are provided.
(TIF)

S9 Fig 1-bromobutane interaction map with LinB surface. For each of the binding sites a histogram of interactions with particular residues and the cumulative interaction time (CT) are provided.
(TIF)

S10 Fig 1-iodopropane interaction map with LinB surface. For each of the binding sites a histogram of interactions with particular residues and the cumulative interaction time (CT) are provided.
(TIF)

S11 Fig 1,2 –dibromoethane interaction map with LinB surface. For each of the binding sites a histogram of interactions with particular residues and the cumulative interaction time (CT) are provided.
(TIF)

S12 Fig 1,2 –dibromopropane interaction map with the LinB surface. For each of the binding sites a histogram of interactions with particular residues and the cumulative interaction time (CT) are provided.
(TIF)

S13 Fig Bromocyclohexane interaction map with the LinB surface. For each of the binding sites a histogram of interactions with particular residues and the cumulative interaction time (CT) are provided.
(TIF)

S14 Fig Chlorocyclohexane interaction map with the LinB surface. For each of the binding sites a histogram of interactions with particular residues and the cumulative interaction time (CT) are provided.
(TIF)

S15 Fig Western blot confirmation of the 6xHis-tag motif in the proteins expressed from the plasmids containing LinB Wild Type and its mutants. (TIF)

S16 Fig Electrophoretic confirmation of the purity and molecular mass of the proteins expressed from the plasmids containing LinB Wild Type and its mutants. (TIF)

S17 Fig Circular dichroism spectra for LinB WT and eight mutants. (TIF)

S18 Fig The analysis of the influence of mutation on the thermostability of the LinB protein. (TIF)

S1 Table Results of FoldX calculations of Gibbs free energy of mutants for the selected positions. The mutants selected for experimental verification are framed and marked bold. Column names indicate positions in protein structure and row names indicate substituted amino acids.
(TIF)

S2 Table Summary table of experimental and *in silico* results. Columns from the left: “LinB”–which mutant, “Change of thermal stability”—Experimentally measured change in thermal stability in comparison to LinB WT, “Gibbs free energy [kcal/mol]”—Gibbs free energy calculated with FoldX, “Change of activity–chlorohexane”—Change of activity experimentally measured with chlorohexane in comparison to LinB WT, “Binding sites-chlorohexane 1–9”–Total interaction time (sum of interaction time of all residues in a given binding site in the course of 10 replicas 100 ns MD simulations with chlorohexane), “Change of activity–bromocyclohexane”—Change of activity experimentally measured with bromocyclohexane in comparison to LinB WT, “Binding sites- bromocyclohexane 1–9”–Total interaction time (sum of interaction time of all residues in a given binding site in the course of 10 replicas 100 ns MD simulations with bromocyclohexane).
(TIF)

S3 Table The yield and predicted content of the secondary structures in the LinB Wild Type (WT) and its mutants. The data was calculated based on their circular dichroism spectra with DichroWeb platform and CDSSTR algorithm.
(TIF)

S4 Table Microorganism strains and plasmids used in the experimental studies. (TIF)

S1 Graphical abstract (TIF)
