# Supplementary figures and images for: Transient binding sites at the surface of haloalkane dehalogenase LinB as locations for fine-tuning enzymatic activity
Source: PLoS One. 2023 Feb 24;18(2):e0280776. doi: 10.1371/journal.pone.0280776 (PMC9956002; doi:10.1371/journal.pone.0280776)

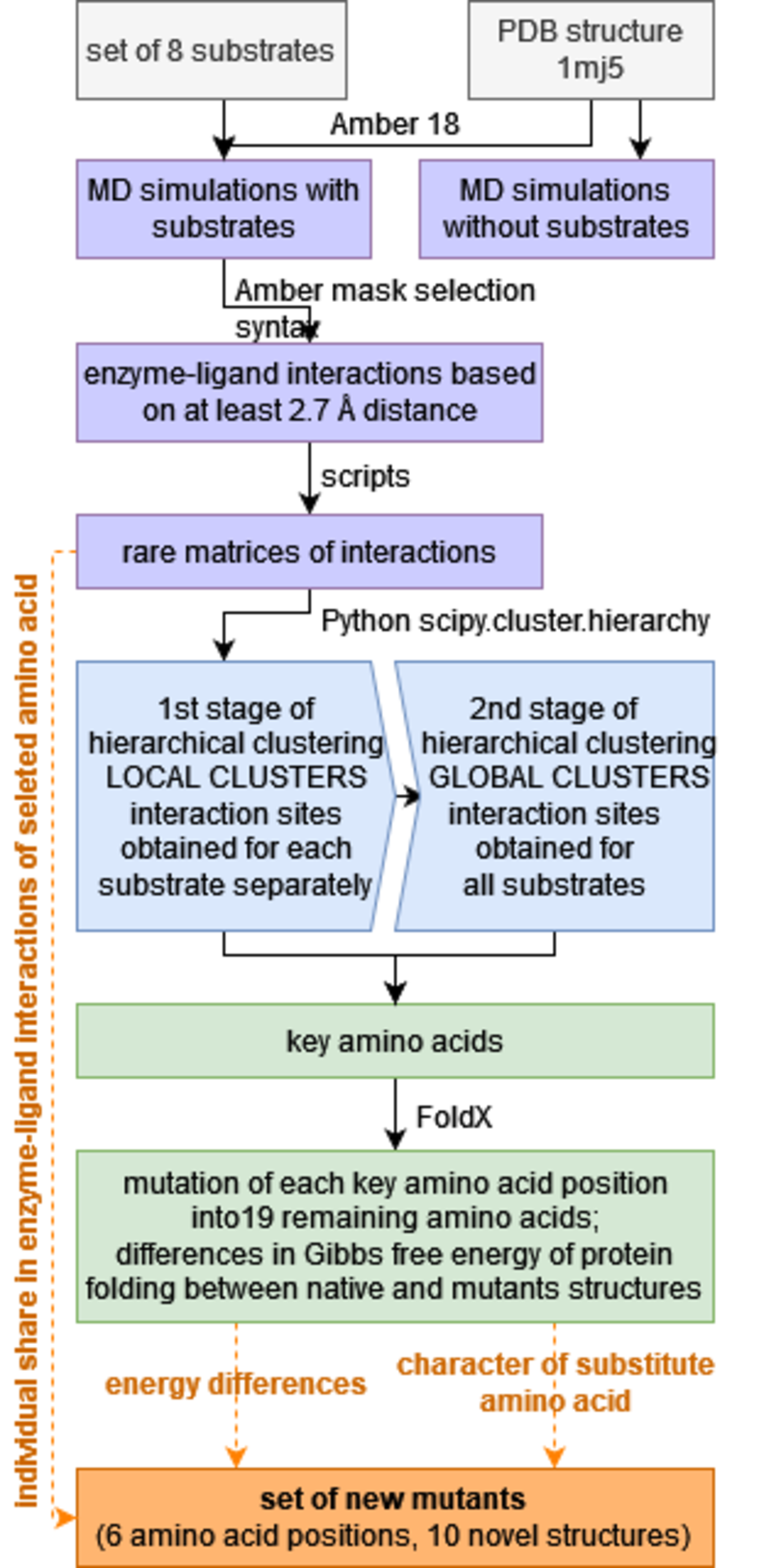

Supplement: S1 Fig — (TIF) [file pone.0280776.s001.tif]

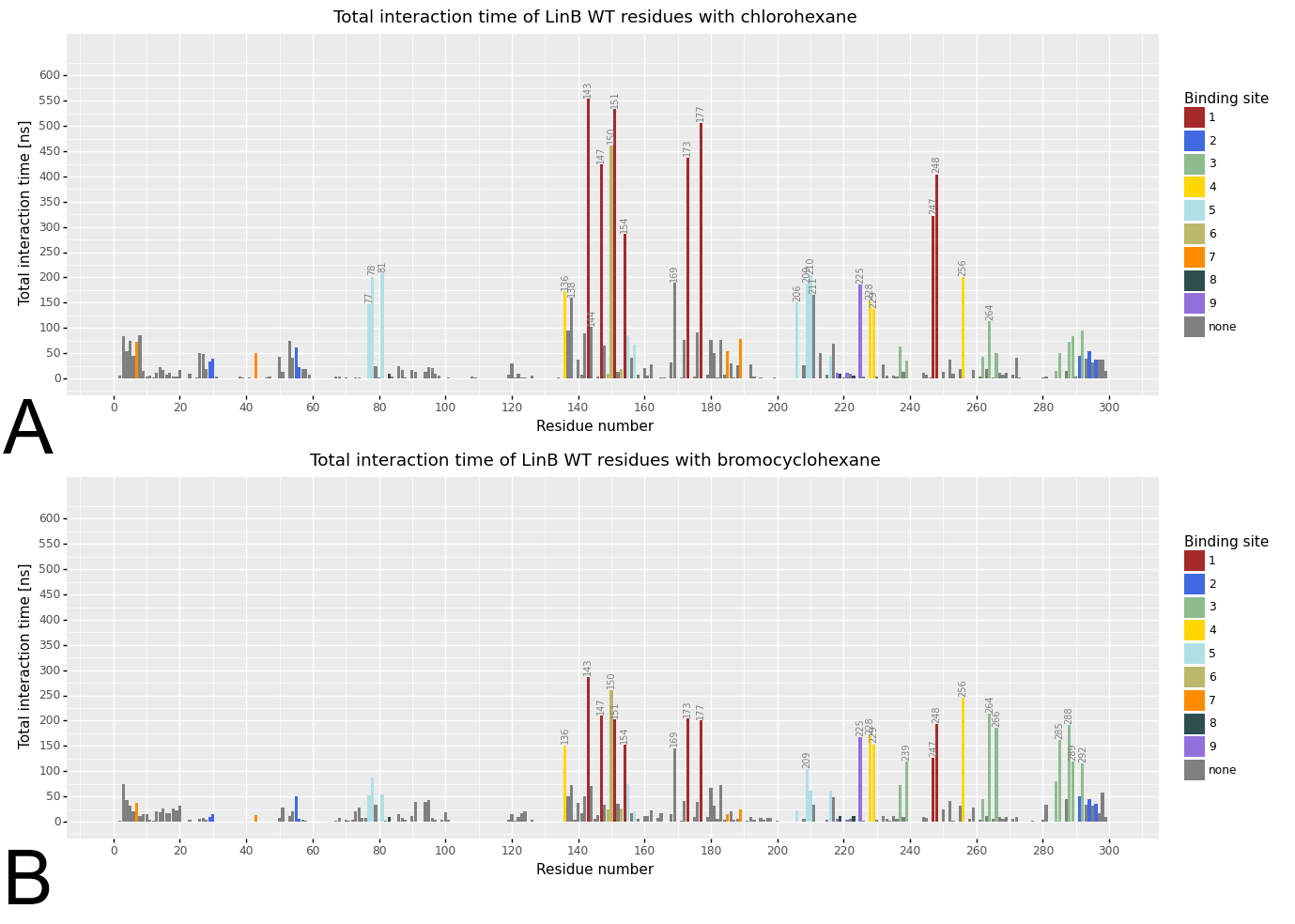

Supplement: S2 Fig — Histogram of LinB WT interaction times of residues with substrate: A) chlorohexane, B) bromocyclohexane. The interaction times were a sum of interactions of all 10 replica 100 ns simulations of LinB WT with a given substrate. (TIF) [file pone.0280776.s002.tif]

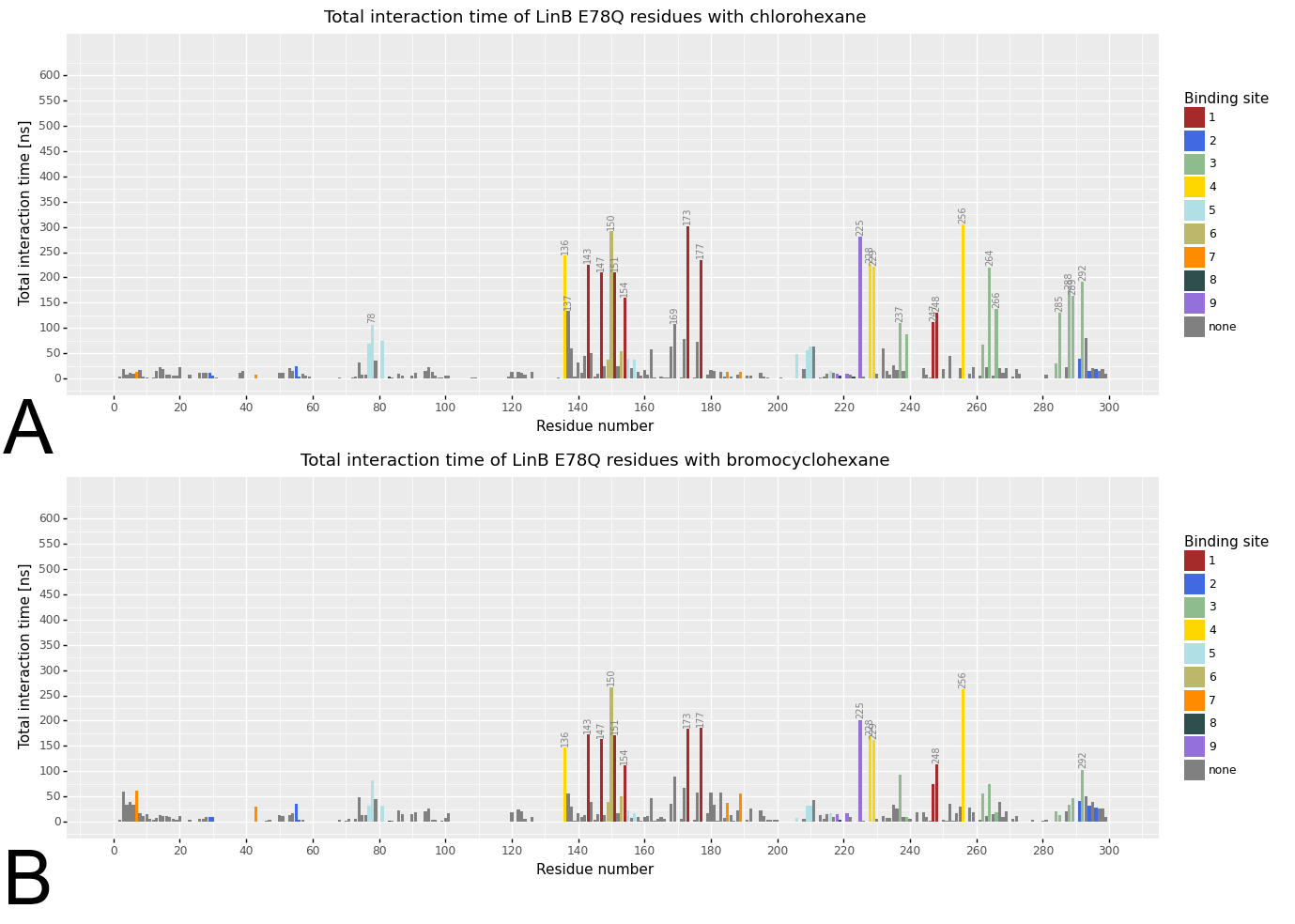

Supplement: S3 Fig — Histogram of LinB E78Q interaction times of residues with substrate: A) chlorohexane, B) bromocyclohexane. The interaction times were a sum of interactions of all 10 replica 100 ns simulations of LinB E78Q with a given substrate. (TIF) [file pone.0280776.s003.tif]

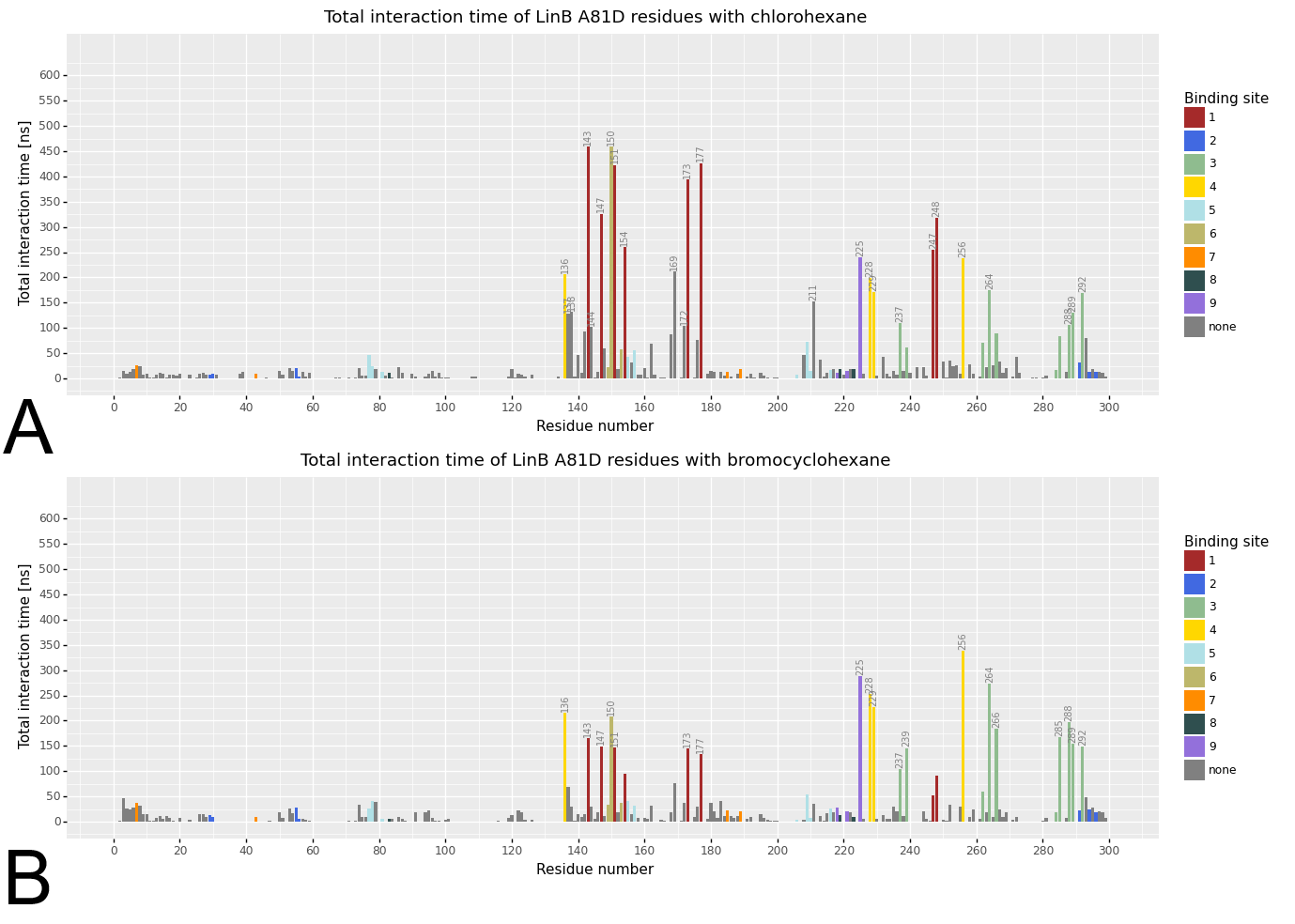

Supplement: S4 Fig — Histogram of LinB A81D interaction times of residues with substrate: A) chlorohexane, B) bromocyclohexane. The interaction times were a sum of interactions of all 10 replica 100 ns simulations of LinB A81D with a given substrate. (TIF) [file pone.0280776.s004.tif]

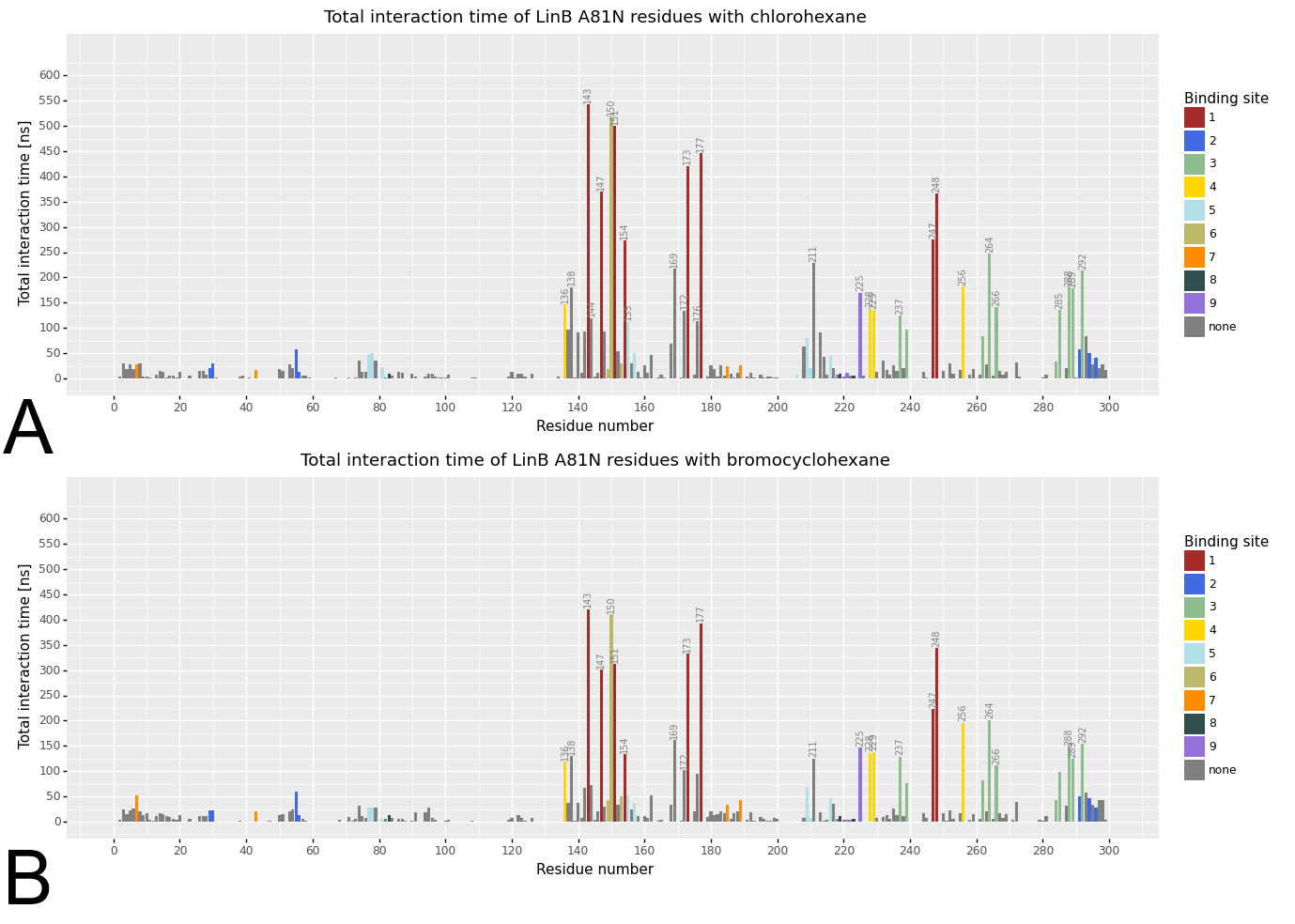

Supplement: S5 Fig — Histogram of LinB A81N interaction times of residues with substrate: A) chlorohexane, B) bromocyclohexane. The interaction times were a sum of interactions of all 10 replica 100 ns simulations of LinB A81N with a given substrate. (TIF) [file pone.0280776.s005.tif]

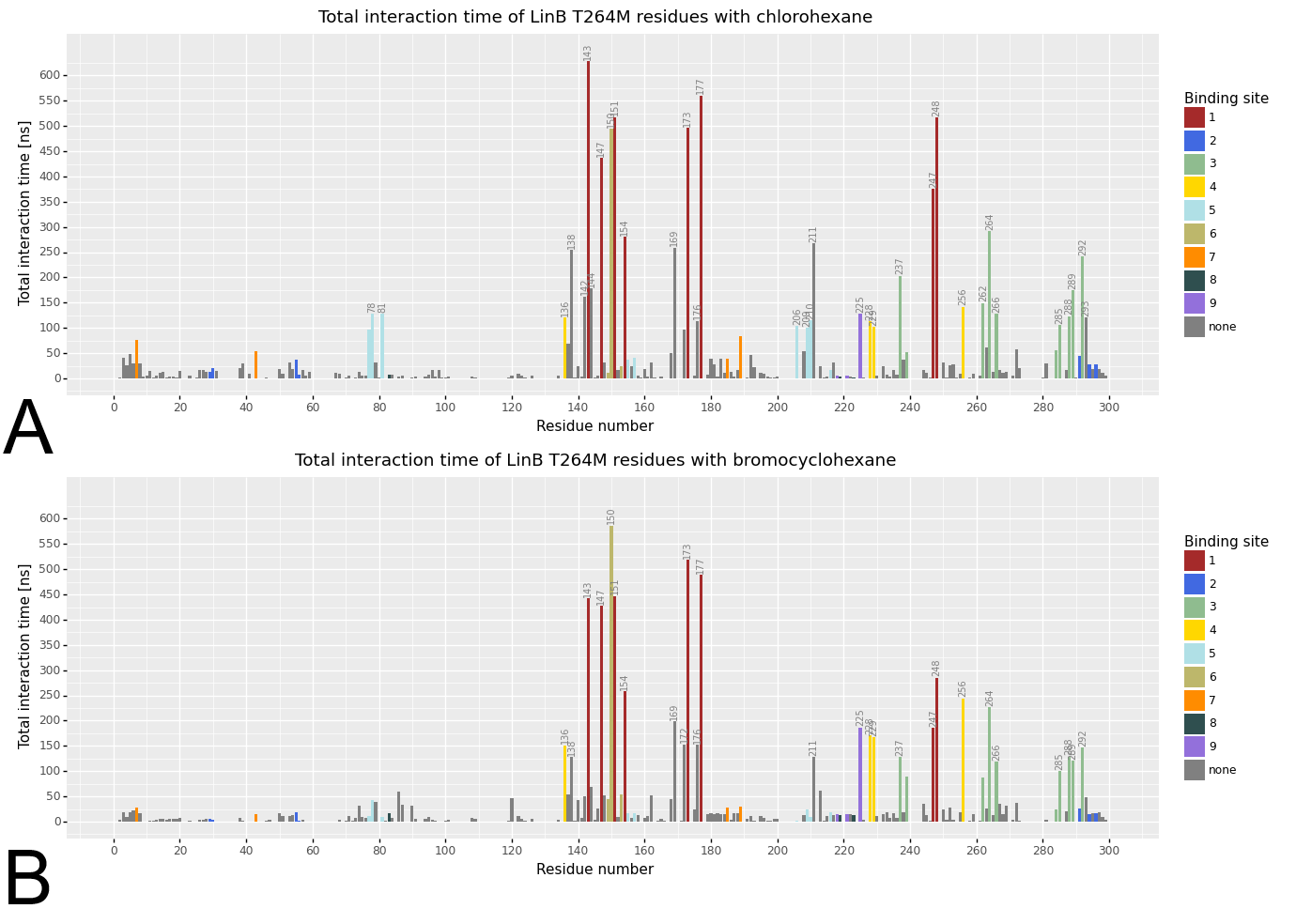

Supplement: S6 Fig — Histogram of LinB T264M interaction times of residues with substrate: A) chlorohexane, B) bromocyclohexane. The interaction times were a sum of interactions of all 10 replica 100 ns simulations of LinB T264M with a given substrate. (TIF) [file pone.0280776.s006.tif]

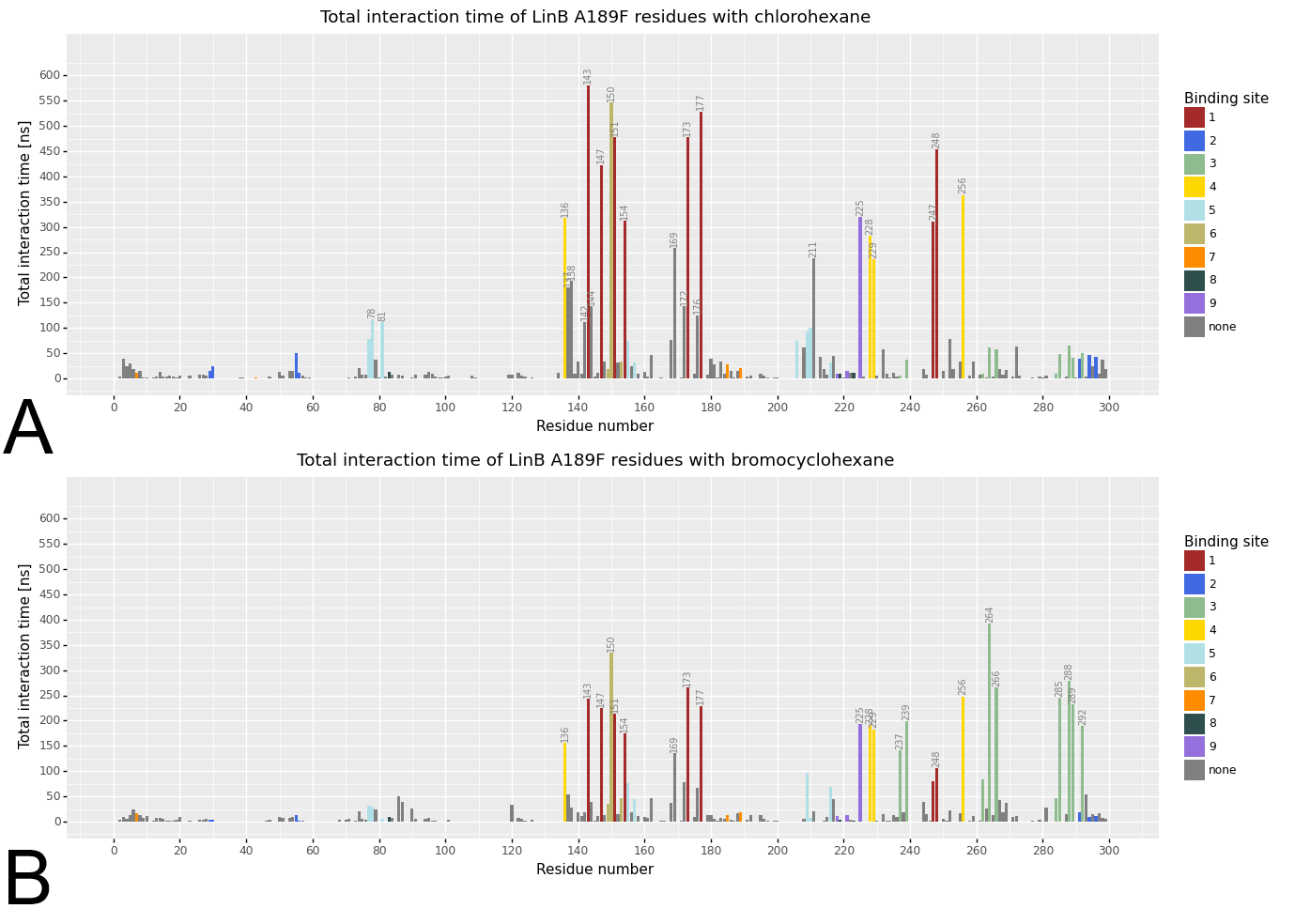

Supplement: S7 Fig — Histogram of LinB A189F interaction times of residues with substrate: A) chlorohexane, B) bromocyclohexane. The interaction times were a sum of interactions of all 10 replica 100 ns simulations of LinB A189F with a given substrate. (TIF) [file pone.0280776.s007.tif]

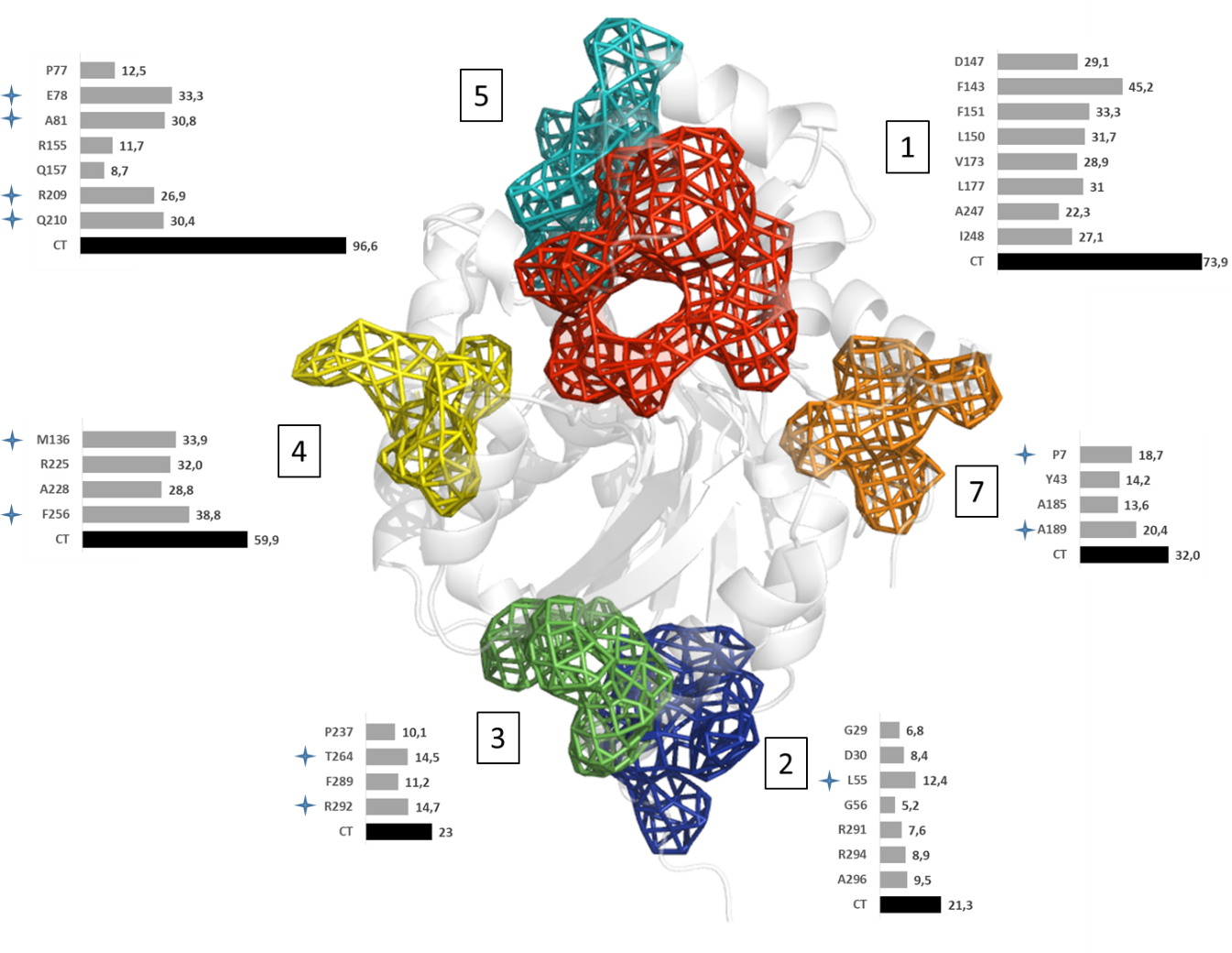

Supplement: S8 Fig — For each of the binding sites a histogram of interactions with particular residues and the cumulative interaction time (CT) are provided. (TIF) [file pone.0280776.s008.tif]

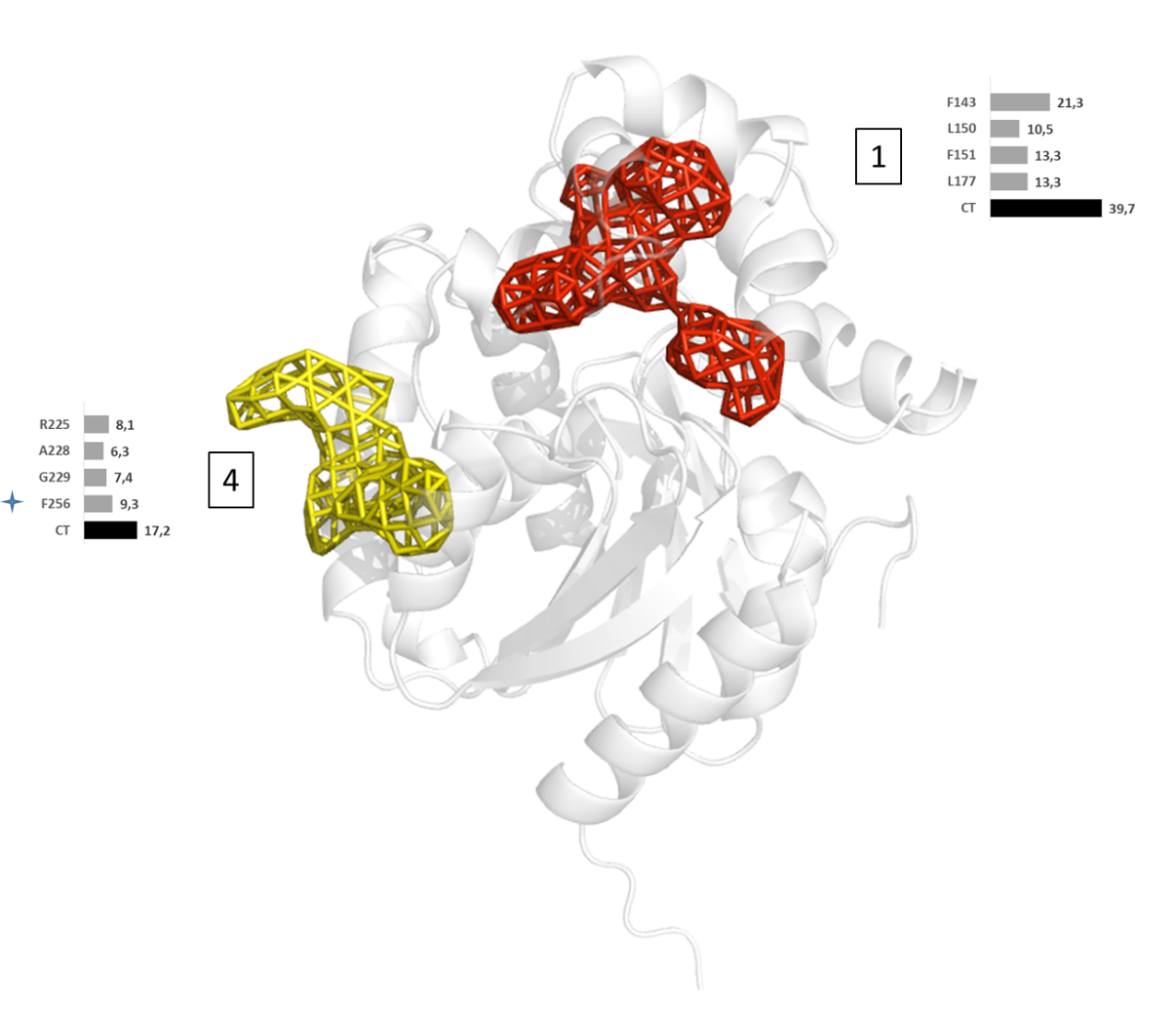

Supplement: S9 Fig — For each of the binding sites a histogram of interactions with particular residues and the cumulative interaction time (CT) are provided. (TIF) [file pone.0280776.s009.tif]

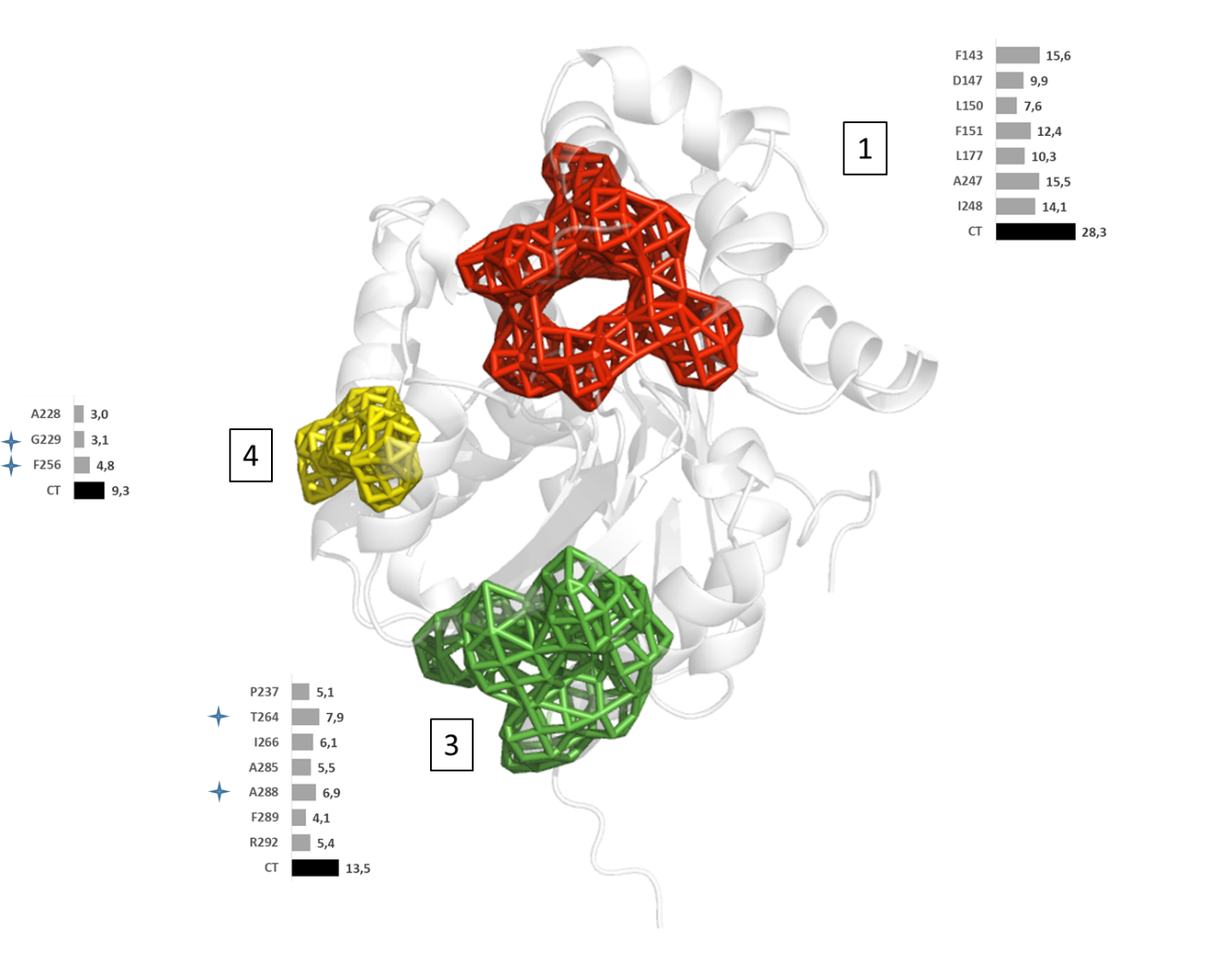

Supplement: S10 Fig — For each of the binding sites a histogram of interactions with particular residues and the cumulative interaction time (CT) are provided. (TIF) [file pone.0280776.s010.tif]

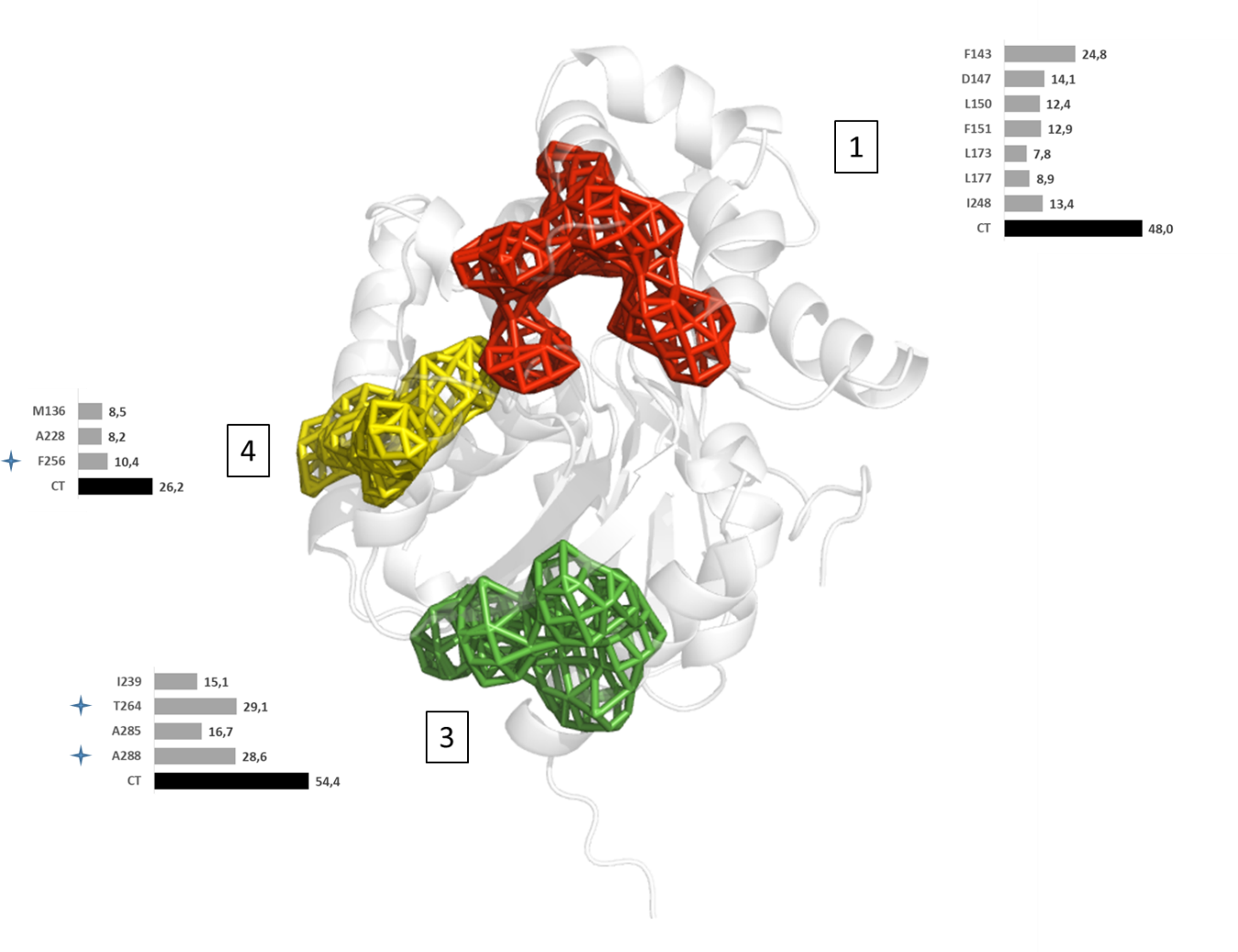

Supplement: S11 Fig — For each of the binding sites a histogram of interactions with particular residues and the cumulative interaction time (CT) are provided. (TIF) [file pone.0280776.s011.tif]

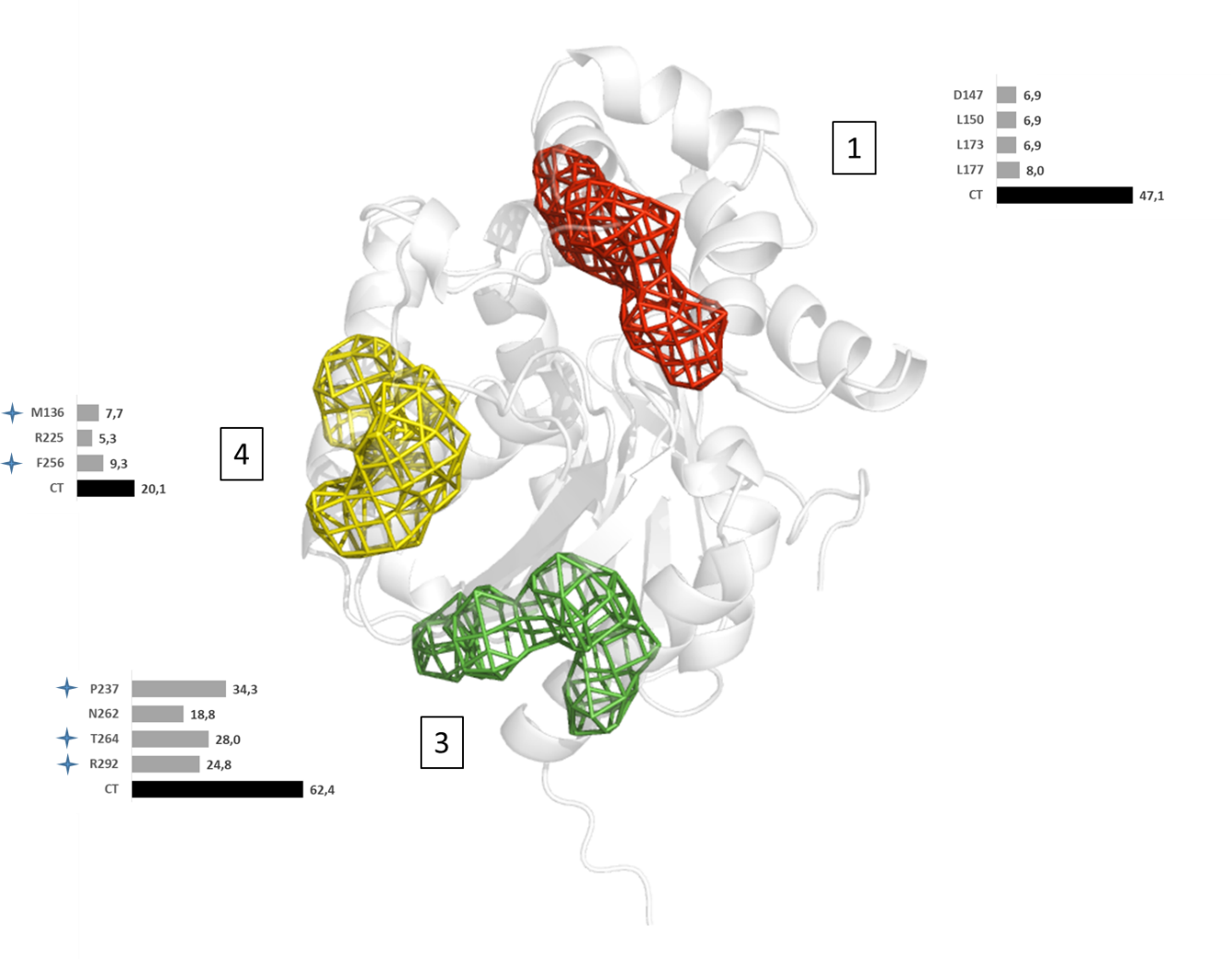

Supplement: S12 Fig — For each of the binding sites a histogram of interactions with particular residues and the cumulative interaction time (CT) are provided. (TIF) [file pone.0280776.s012.tif]

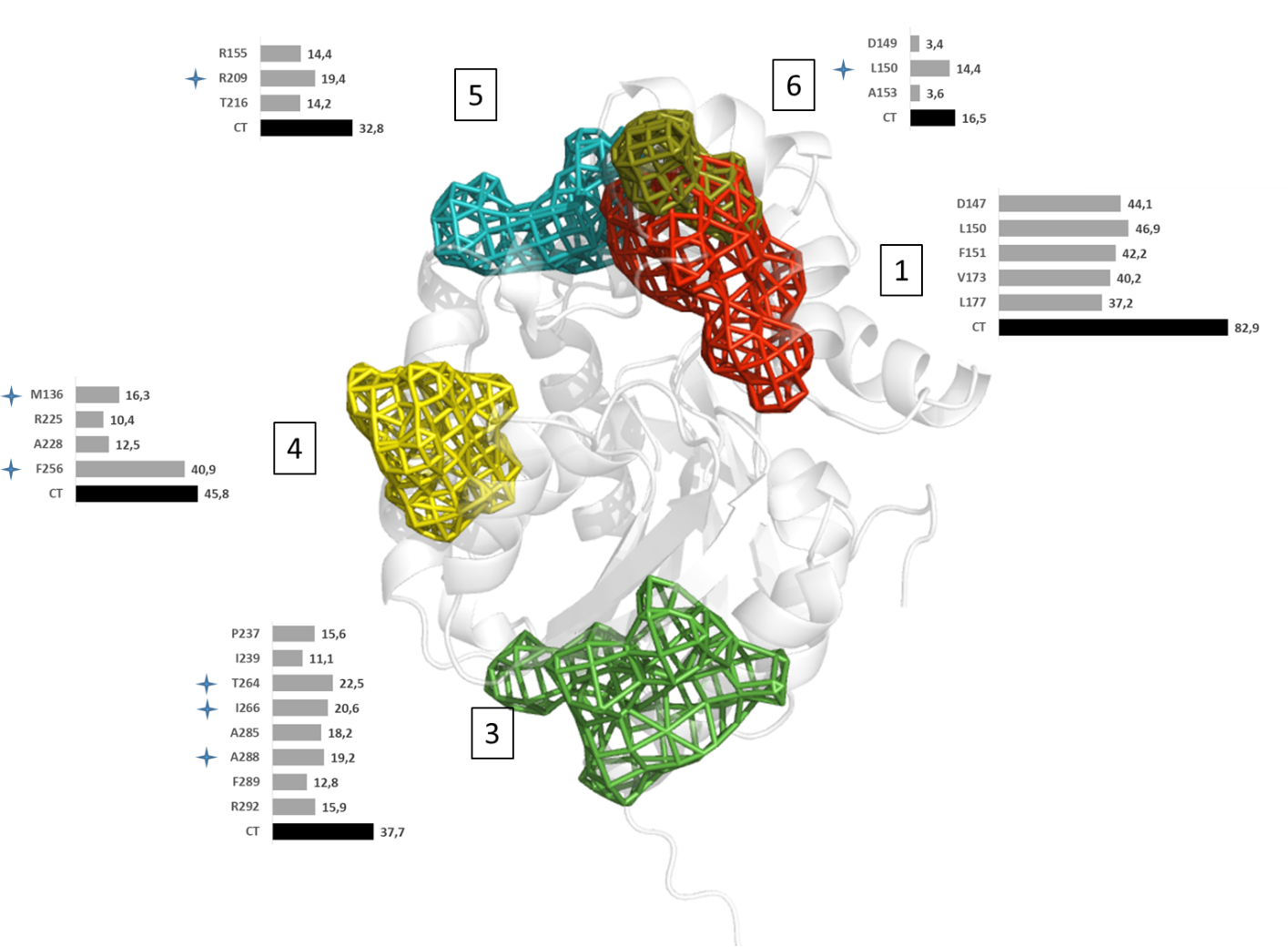

Supplement: S13 Fig — For each of the binding sites a histogram of interactions with particular residues and the cumulative interaction time (CT) are provided. (TIF) [file pone.0280776.s013.tif]

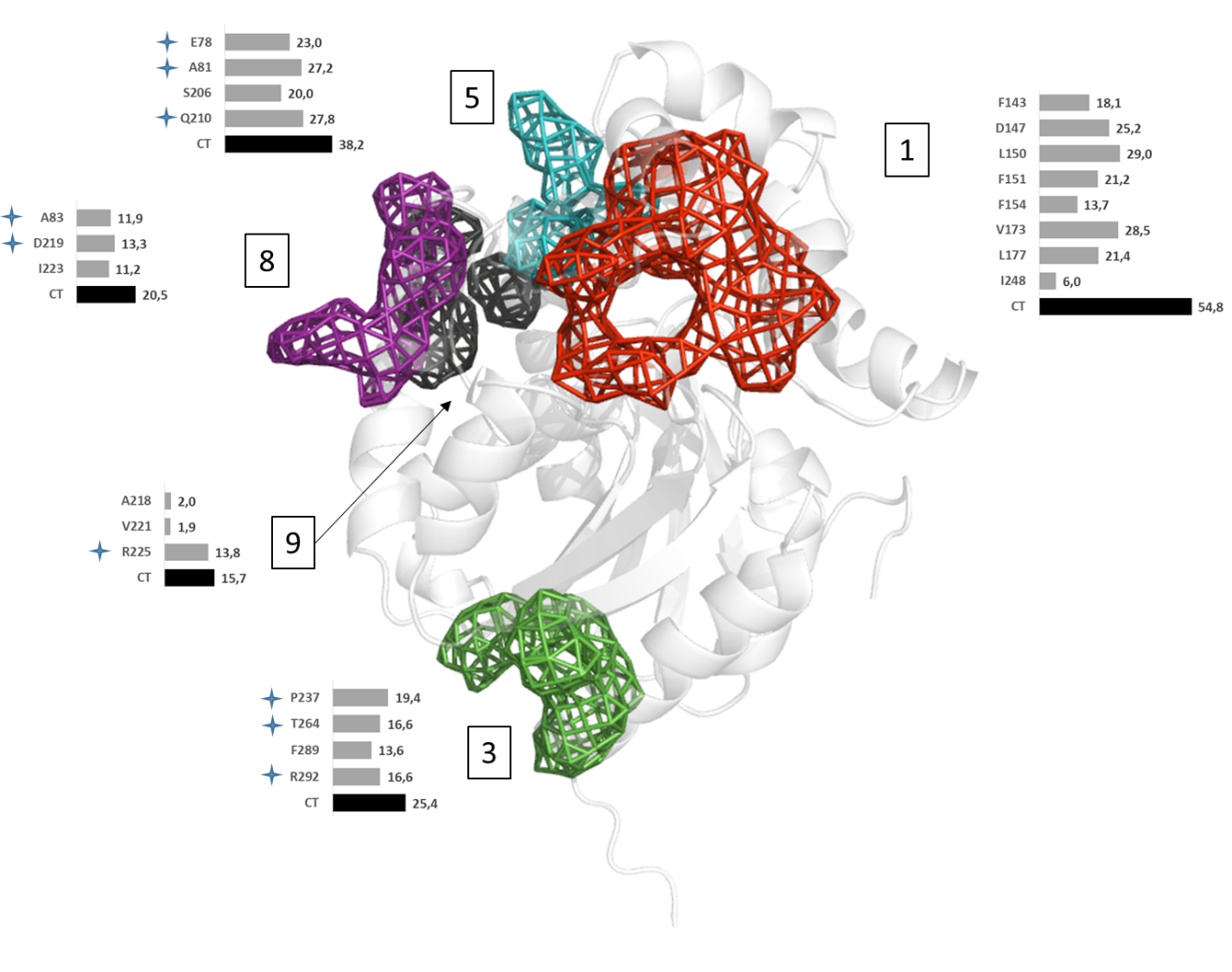

Supplement: S14 Fig — For each of the binding sites a histogram of interactions with particular residues and the cumulative interaction time (CT) are provided. (TIF) [file pone.0280776.s014.tif]

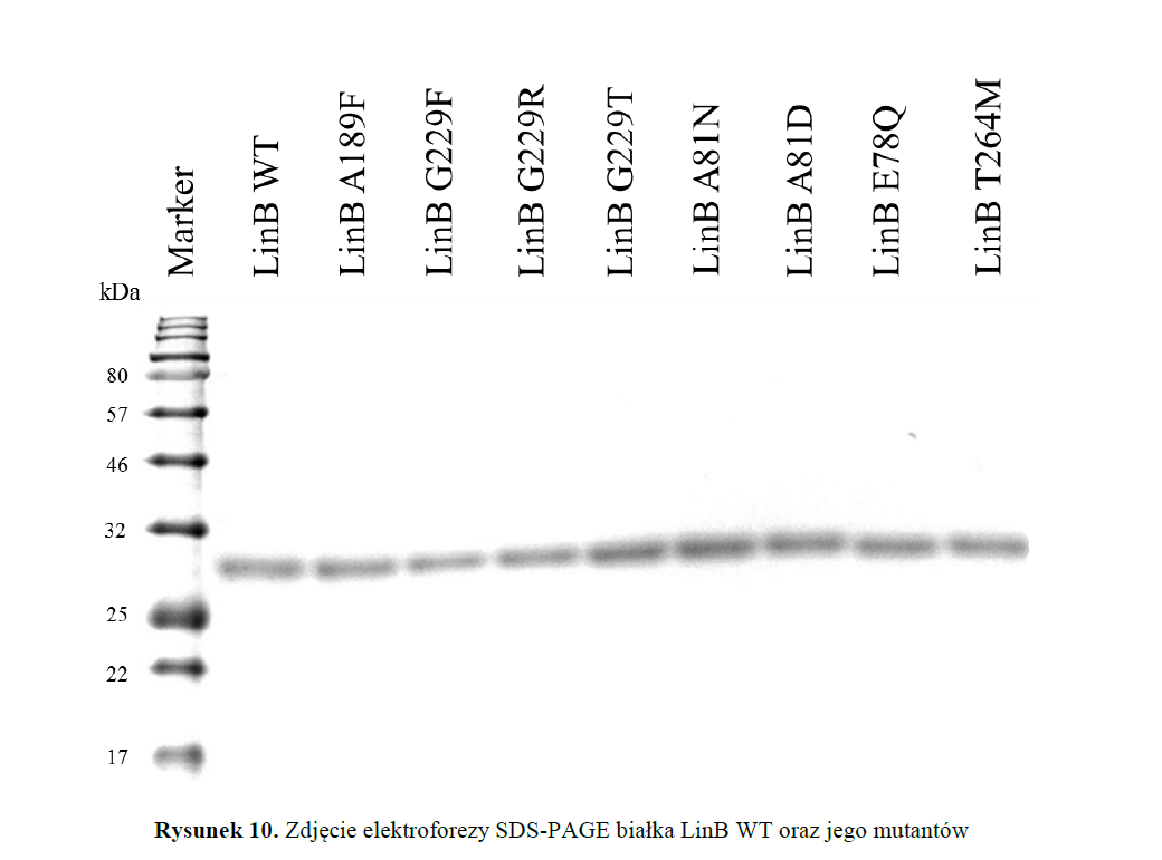

Supplement: S15 Fig — (TIF) [file pone.0280776.s015.tif]

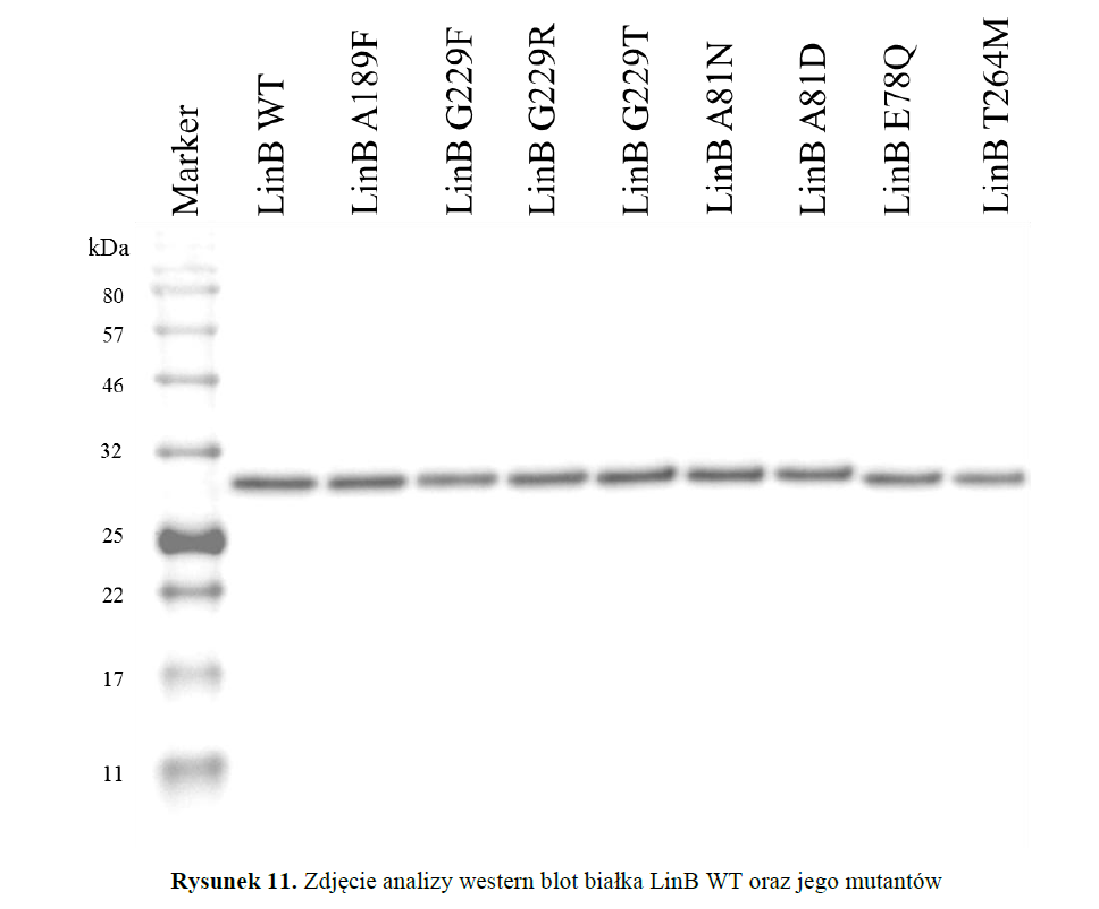

Supplement: S16 Fig — (TIF) [file pone.0280776.s016.tif]

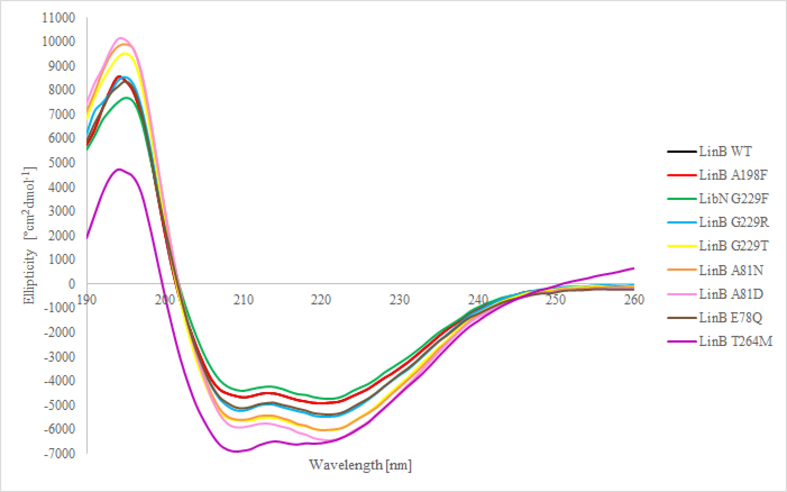

Supplement: S17 Fig — (TIF) [file pone.0280776.s017.tif]

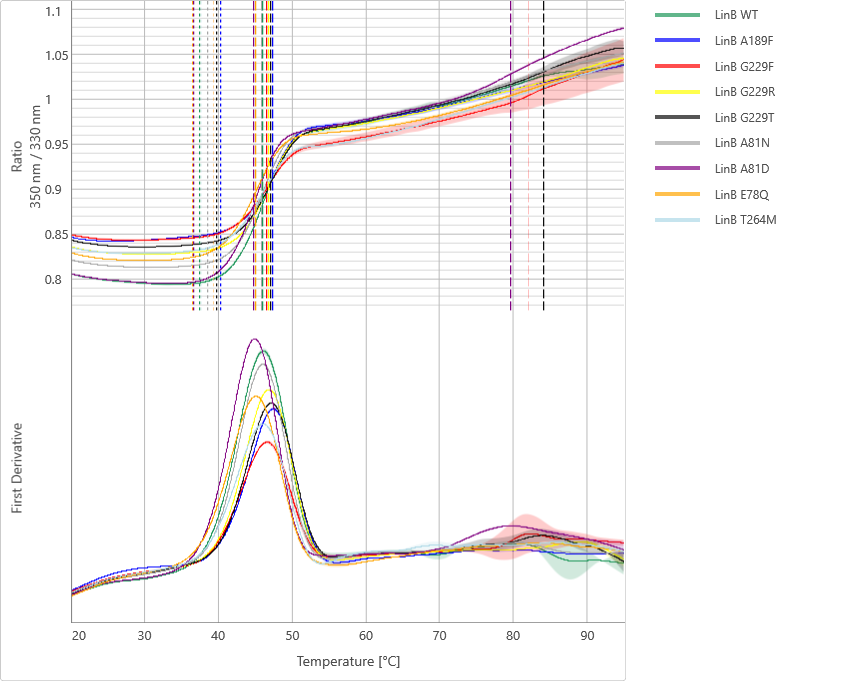

Supplement: S18 Fig — (TIF) [file pone.0280776.s018.tif]

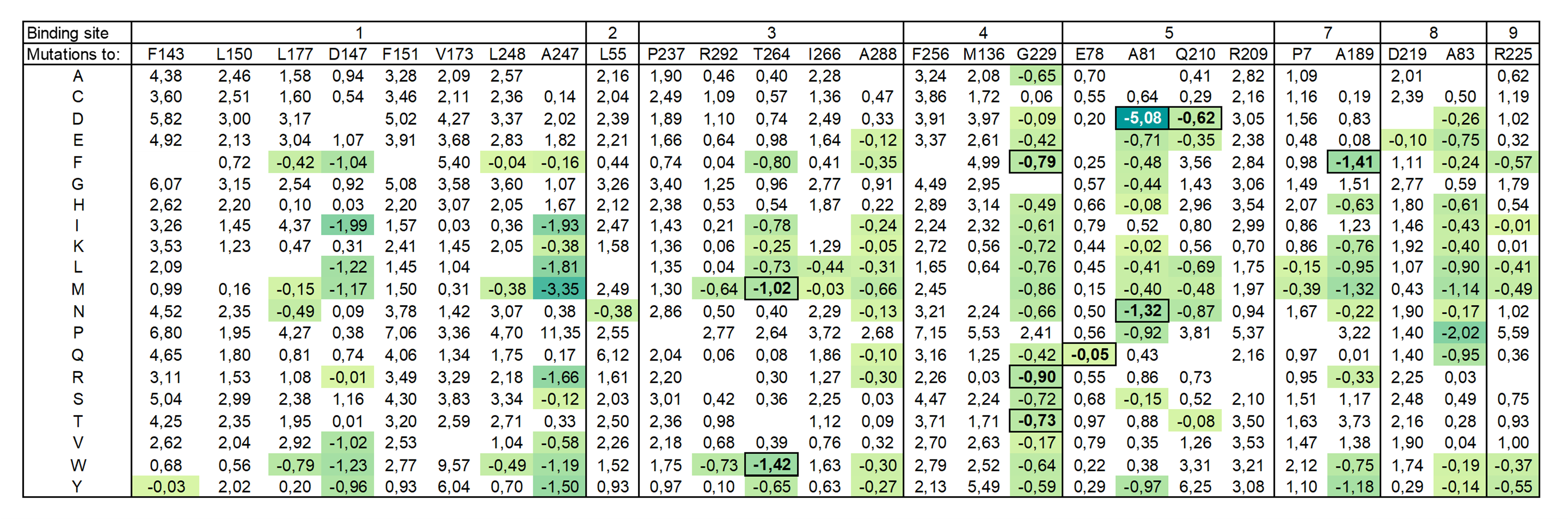

Supplement: S1 Table — The mutants selected for experimental verification are framed and marked bold. Column names indicate positions in protein structure and row names indicate substituted amino acids. (TIF) [file pone.0280776.s019.tif]

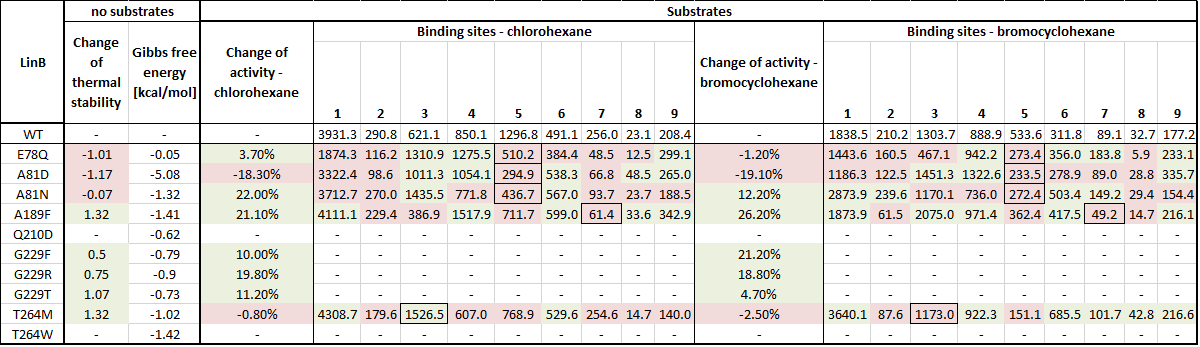

Supplement: S2 Table — Columns from the left: “LinB”–which mutant, “Change of thermal stability”—Experimentally measured change in thermal stability in comparison to LinB WT, “Gibbs free energy [kcal/mol]”—Gibbs free energy calculated with FoldX, “Change of activity–chlorohexane”—Change of activity experimentally measured with chlorohexane in comparison to LinB WT, “Binding sites-chlorohexane 1–9”–Total interaction time (sum of interaction time of all residues in a given binding site in the course of 10 replicas 100 ns MD simulations with chlorohexane), “Change of activity–bromocyclohexane”—Change of activity experimentally measured with bromocyclohexane in comparison to LinB WT, “Binding sites- bromocyclohexane 1–9”–Total interaction time (sum of interaction time of all residues in a given binding site in the course of 10 replicas 100 ns MD simulations with bromocyclohexane). (TIF) [file pone.0280776.s020.tif]

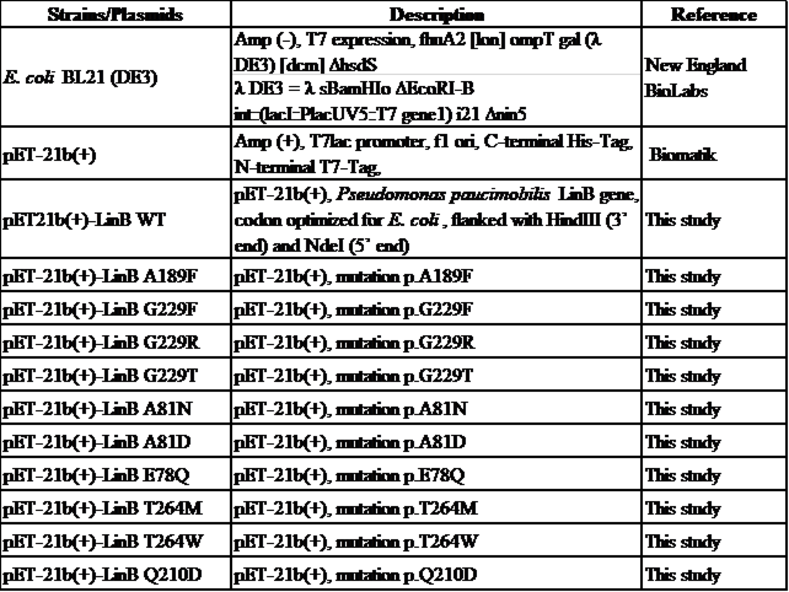

Supplement: S4 Table — (TIF) [file pone.0280776.s022.tif]

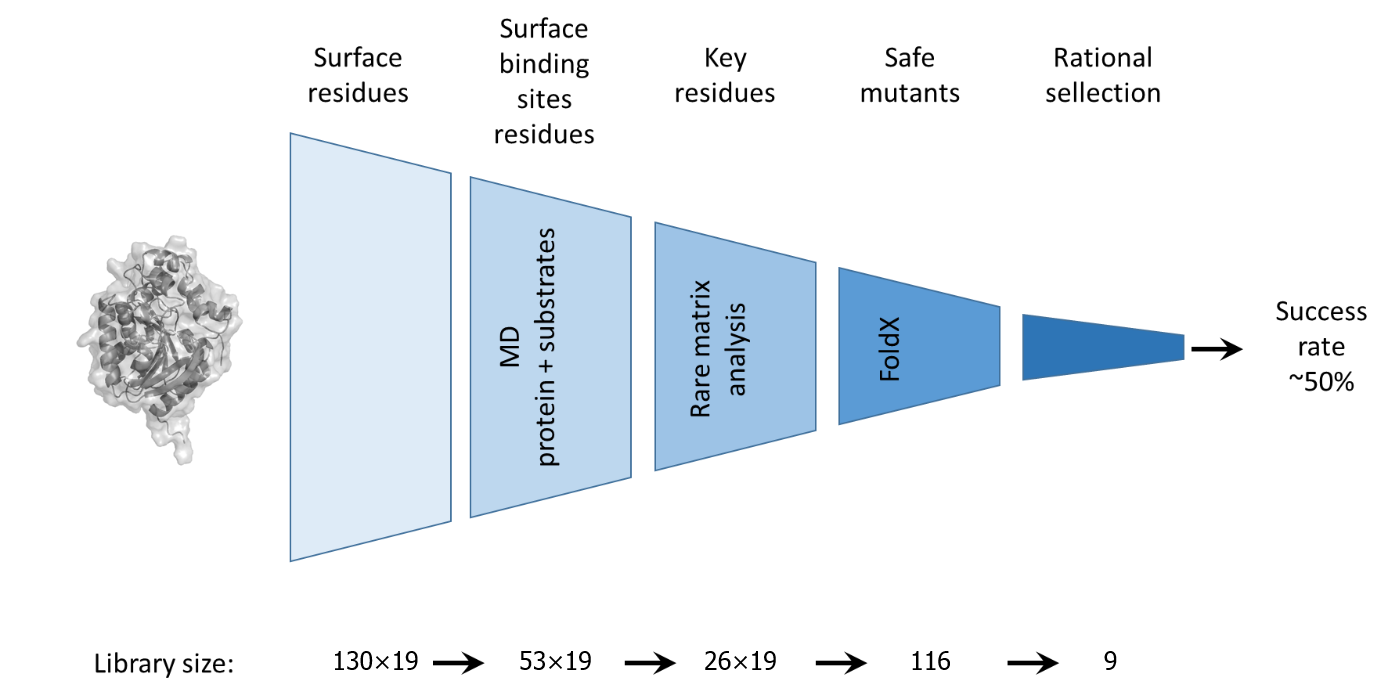

Supplement: S1 Graphical abstract — (TIF) [file pone.0280776.s023.tif]
